# Supplementary material for: Structural Features of Nucleoproteins from the Recently Discovered Orthonairovirus songlingense and Norwavirus beijiense
Source: Int J Mol Sci. 2025 Aug 1;26(15):7445. doi: 10.3390/ijms26157445 (PMC12347420; doi:10.3390/ijms26157445)
Supplement: Supplementary file 1 [file ijms-26-07445-s001.zip › ijms-3759454-supplementary.pdf]

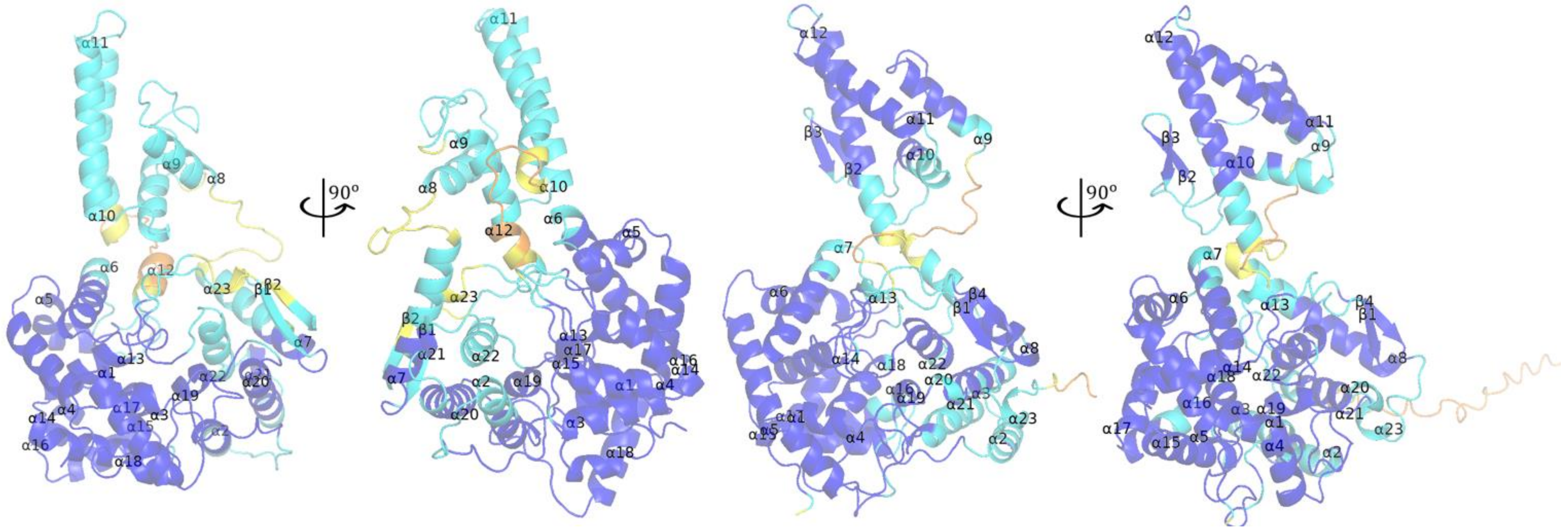

Figure S1. Predicted pLDDT confidence scores of SGLV N (**left**) and BJNV N (**right**) with annotated secondary structure features. The color gradient indicates model pLDDT confidence, while α-helices and β-strands are marked to illustrate protein patterns.

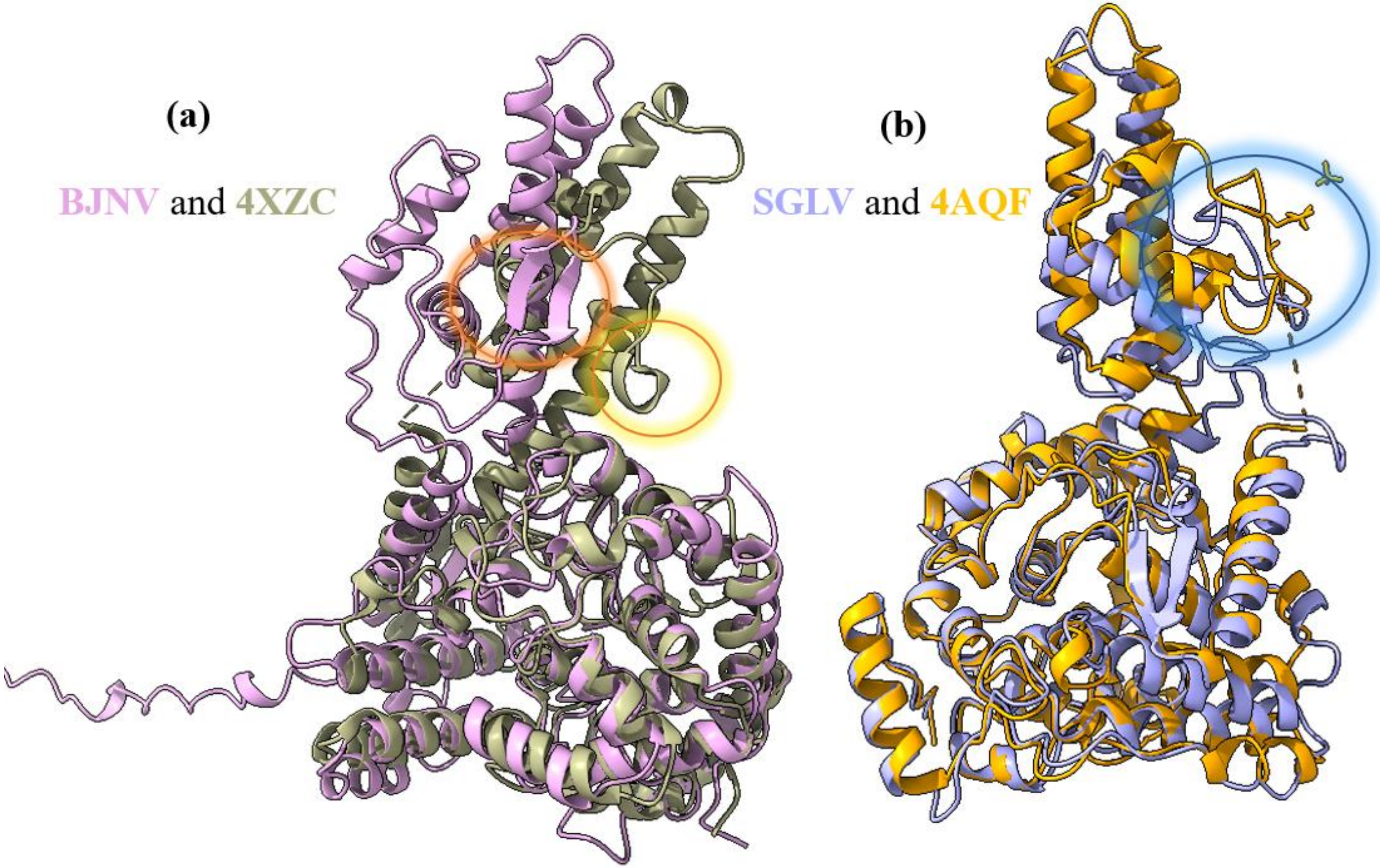

Figure S2. Comparison of nucleoprotein stalk domain structures: **(a)** BJNV N structure compared with PDB ID: 4XZC, showing the presence of a two-stranded β-sheet motif in the stalk domain, consistent with the previously observed β-strand motif in KUPV N (4XZC); **(b)** SGLV N structure compared with PDB ID: 4AQF, demonstrating the absence of this β-sheet motif in the stalk domain, indicating structural divergence in this region.

Table S1. Hydrodynamic parameter predictions calculated based on AlphaFold 3 structures using the HullRad server (http://52.14.70.9/Run_hullrad.html).

	**SGLV N**	**BJNV N**
#Amino Acids	487	556
M (g/mol)	54 137	60 992
v_bar (mL/g)	0.735	0.738
Ro (Anhydrous) (Angstroms)	25.08	26.13
Rg (Anhydrous) (Angstroms)	24.72	30.02
Dmax (Angstroms)	87.62	132.31
Axial Ratio	1.74	2.48
f/fo	1.26	1.46
Dt (cm^2/s)	6.81e-07	5.63e-07
R (Translation) (Angstroms)	31.50	38.08
s20, w (sec)	4.02e-13	3.71e-13
Int. Viscosity (mL/g)	4.21	6.43
Total Hydration (g/g)	0.66	1.31
Spc Vol Hyd Prot (mL/g)	1.40	2.05
ks(non-ideal) (mL/g)	7.82	11.88
kd(non-ideal) (mL/g)	4.64	7.75
Bex (2nd virial) (mL/g) (Excluded volume)	5.82	9.14
Asphericity (from Gyration Tensor)	0.17	0.13
Dr (s^-1)	4.01e+06	1.84e+06
R(Rotation) (Angstroms)	34.23	44.38
tauC (ns) (from R_rotation)	41.55	90.61

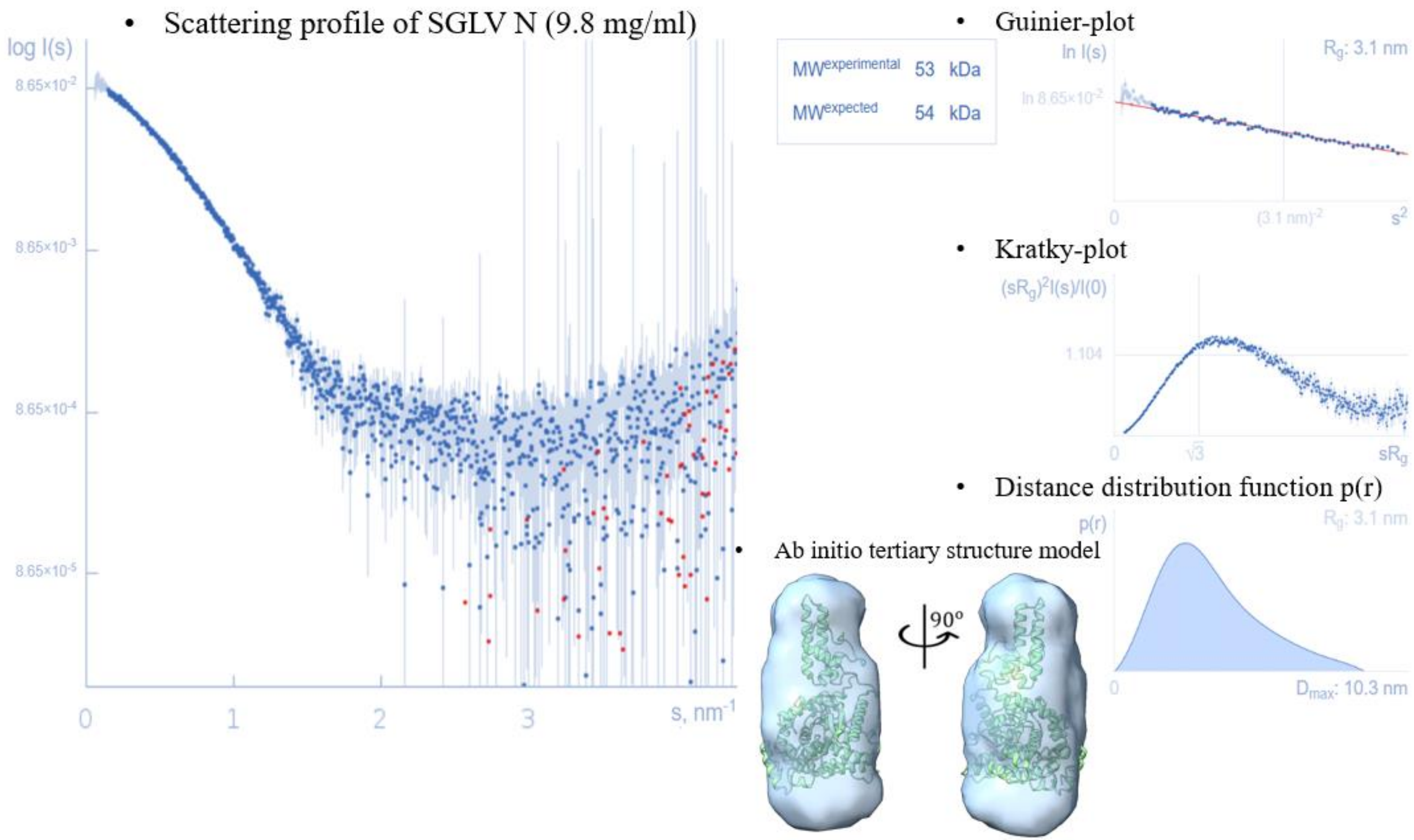

Figure S3. SAXS analysis of full-length SGLV N at concentrations of 9.8 mg/ml, normalized Kratky-plot indicate the intersection point of the lines qRg = $\sqrt{3}$ and $(q\,Rg)^2 \cdot I(q) / I(0) = 1.104$, corresponds to the expected peak position of the Kratky plot for an ideal, compact globular particle.

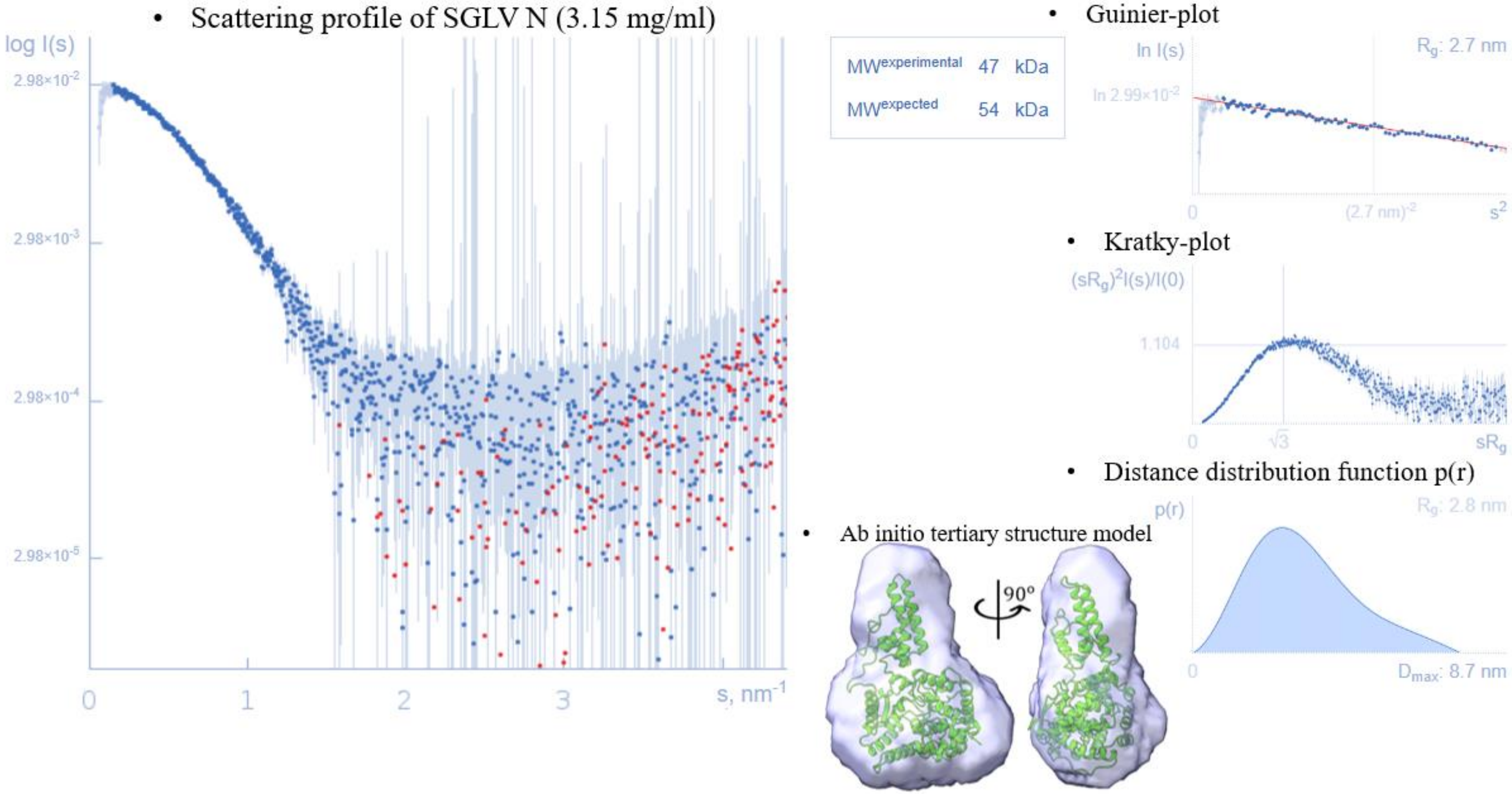

Figure S4. SAXS analysis of full-length SGLV N at concentrations of 3.15 mg/ml, normalized Kratky-plot indicate the intersection point of the lines qRg = $\sqrt{3}$ and $(q\,Rg)^2 \cdot I(q) / I(0) = 1.104$, corresponds to the expected peak position of the Kratky plot for an ideal, compact globular particle.

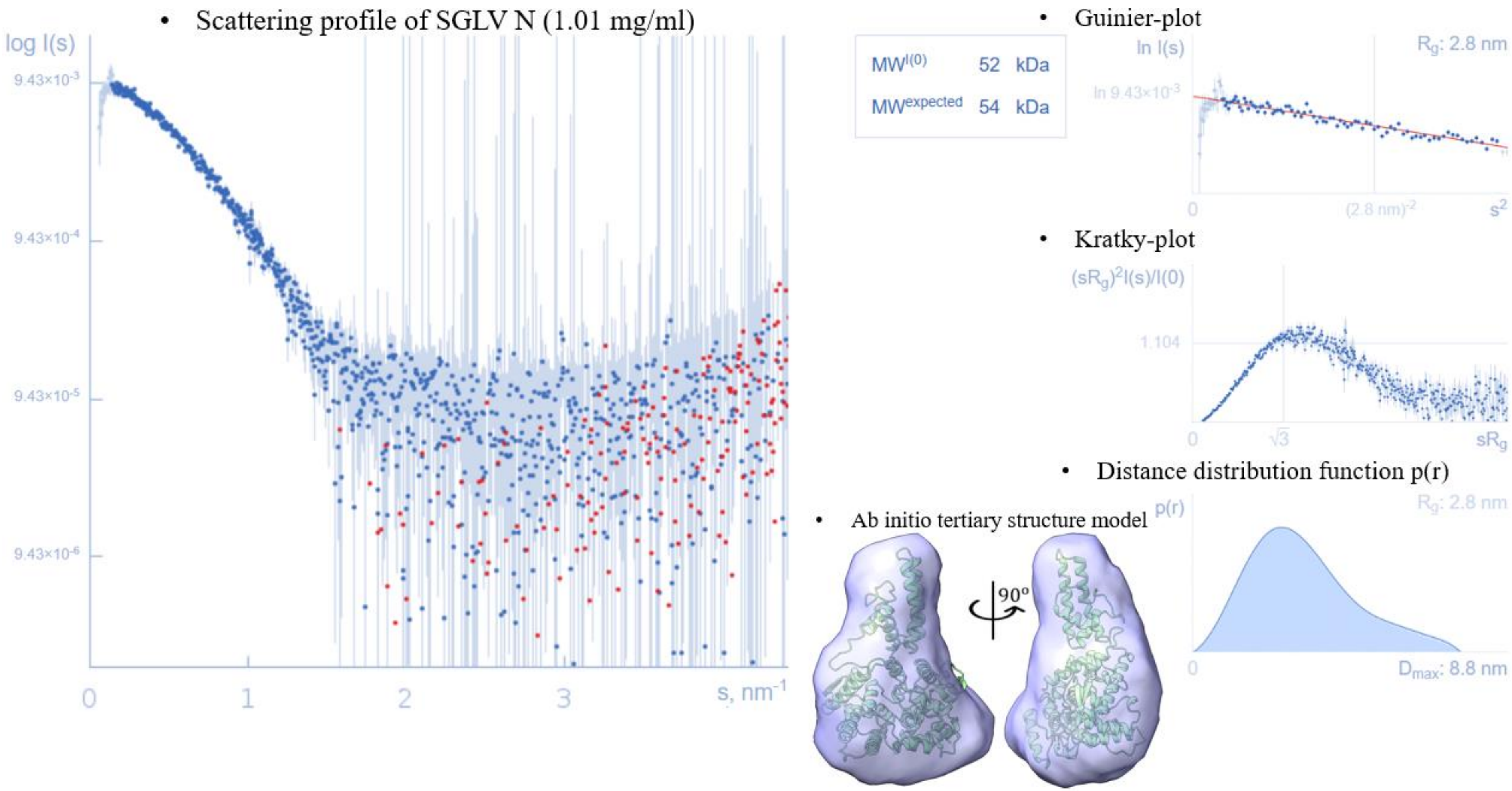

Figure S5. SAXS analysis of full-length SGLV N at concentrations of 1.01 mg/ml, normalized Kratky-plot indicate the intersection point of the lines qRg = $\sqrt{3}$ and $(q\ Rg)^2 \cdot I(q) / I(0) = 1.104$, corresponds to the expected peak position of the Kratky plot for an ideal, compact globular particle.

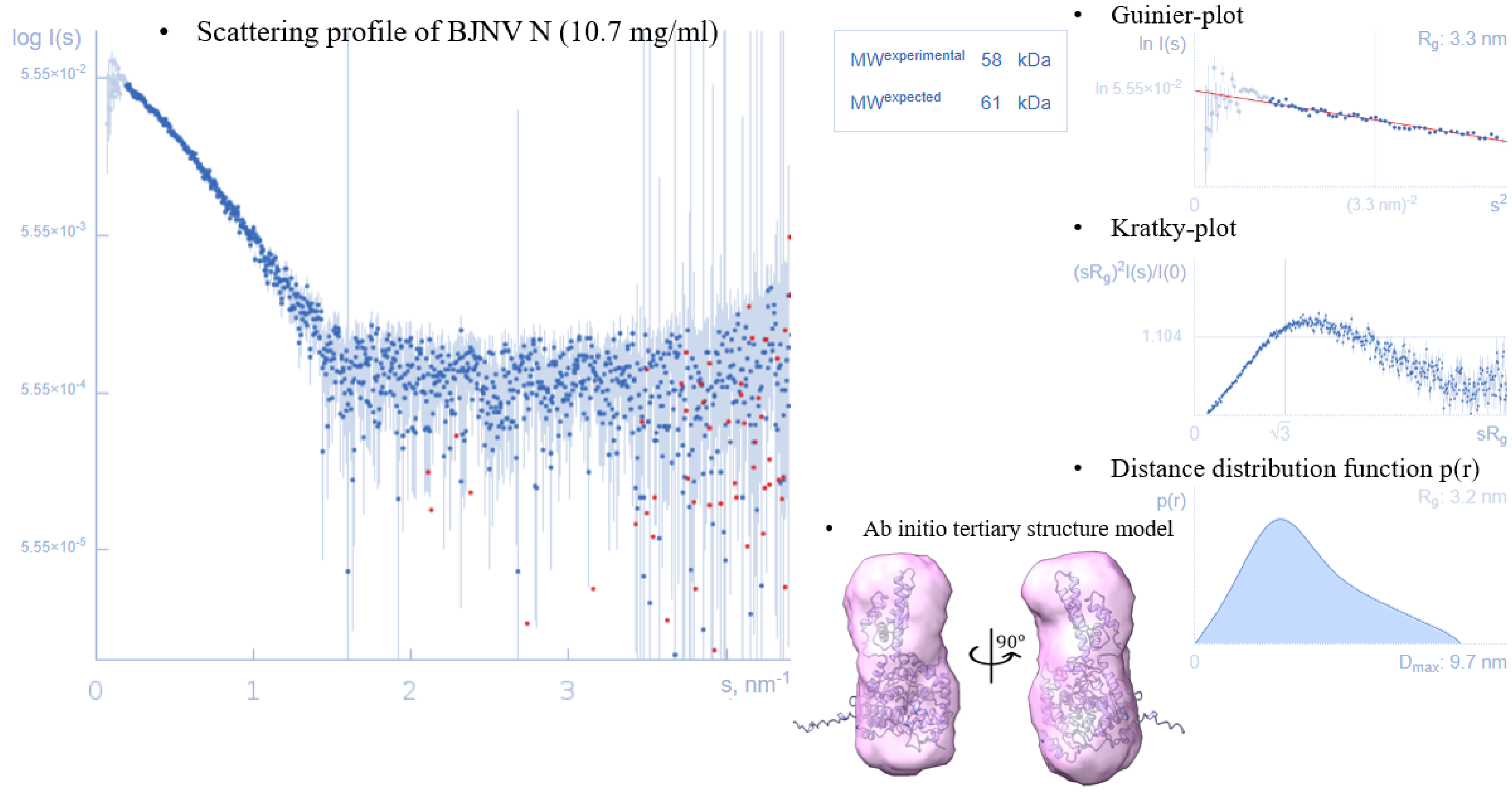

Figure S6. SAXS analysis of full-length BJNV N at concentrations of 10.7 mg/ml, normalized Kratky-plot indicate the intersection point of the lines qRg = $\sqrt{3}$ and $(q\ Rg)^2 \cdot I(q) / I(0) = 1.104$, corresponds to the expected peak position of the Kratky plot for an ideal, compact globular particle.

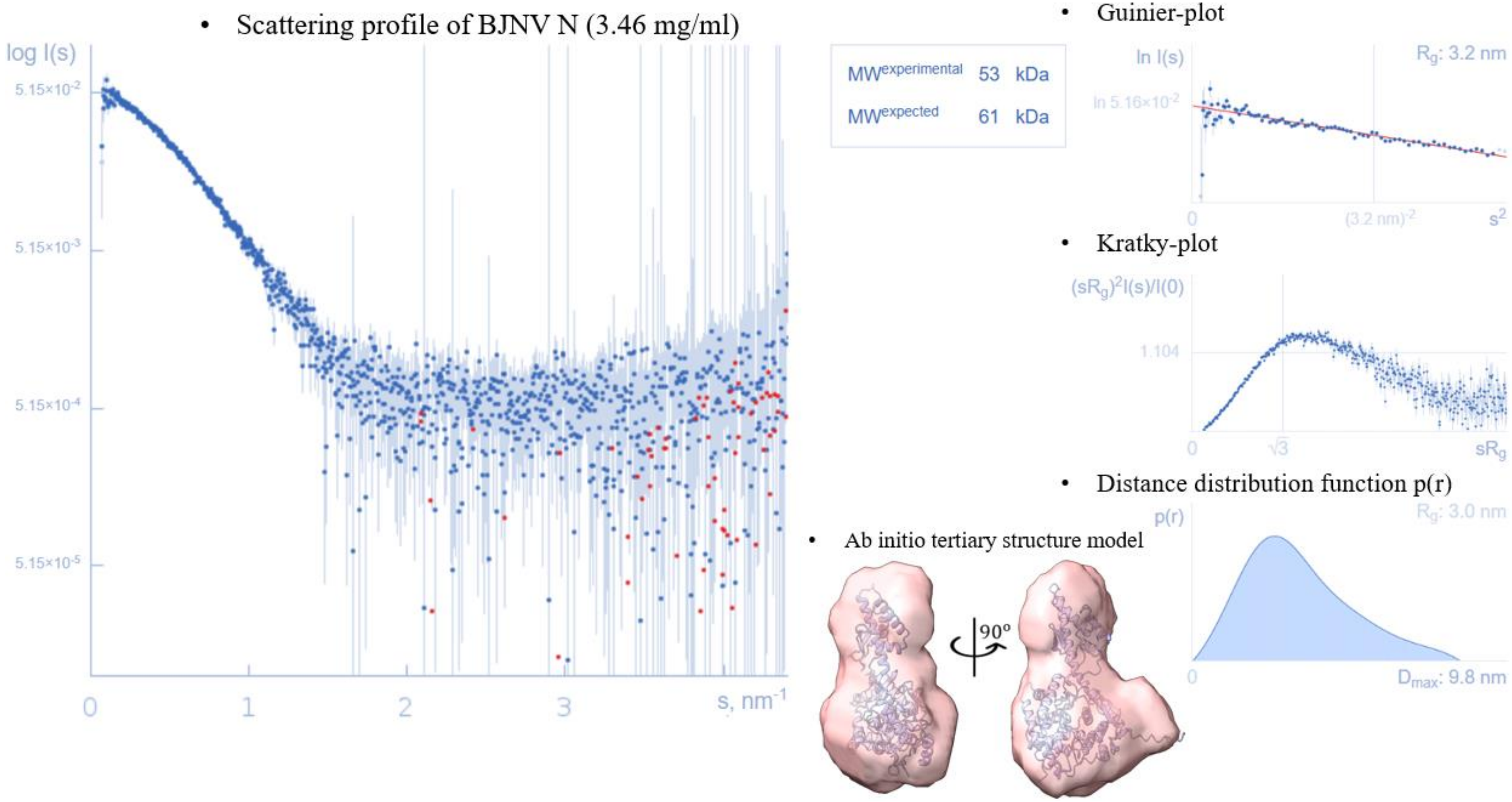

Figure S7. SAXS analysis of full-length BJNV N at concentrations of 3.46 mg/ml, normalized Kratky-plot indicate the intersection point of the lines $qRg = \sqrt{3}$ and $(q\,Rg)^2 \cdot I(q) / I(0) = 1.104$, corresponds to the expected peak position of the Kratky plot for an ideal, compact globular particle.

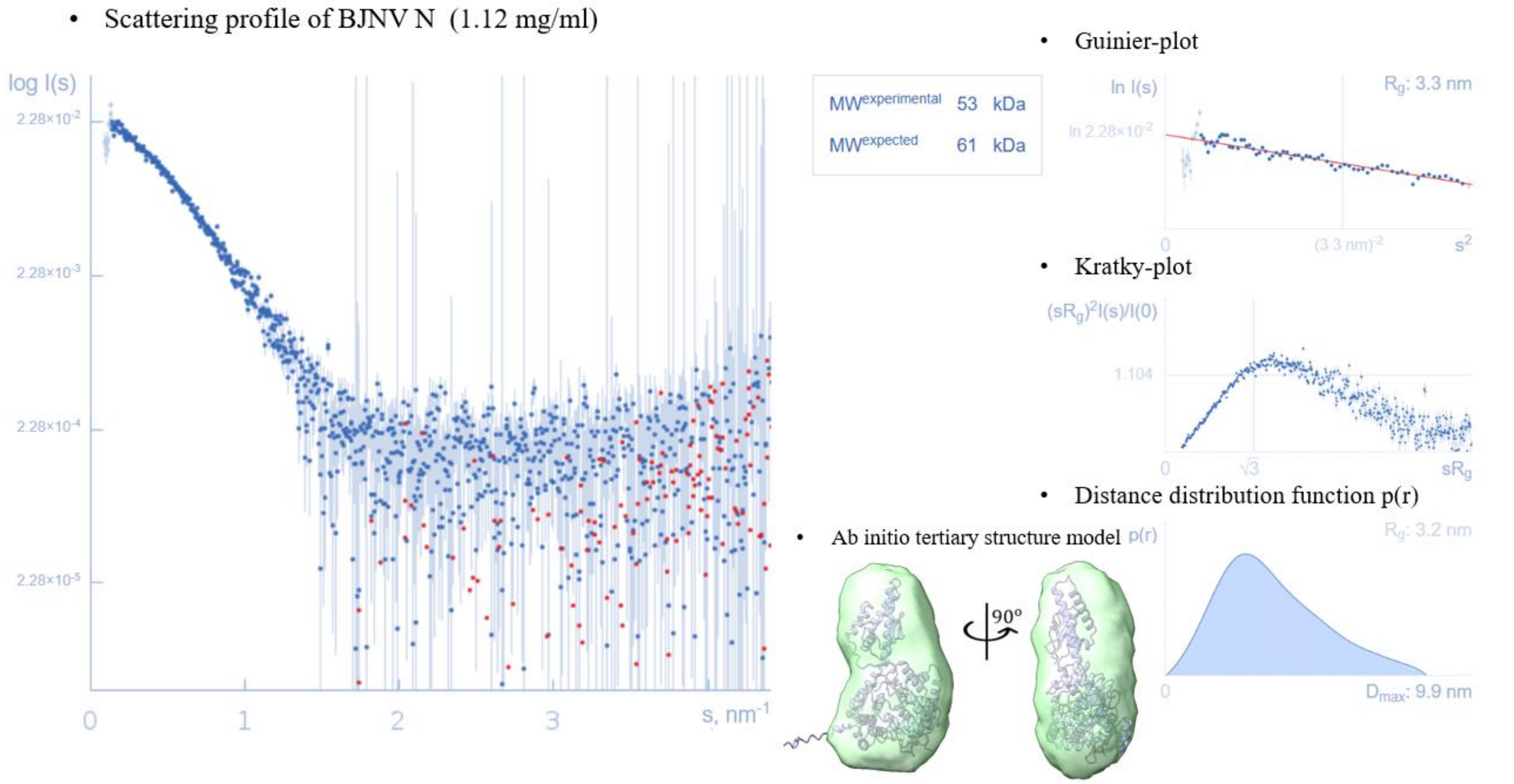

Figure S8. SAXS analysis of full-length BJNV N at concentrations of 1.12 mg/ml, normalized Kratky-plot indicate the intersection point of the lines qRg = $\sqrt{3}$ and $(q\ Rg)^2 \cdot I(q) / I(0) = 1.104$, corresponds to the expected peak position of the Kratky plot for an ideal, compact globular particle.

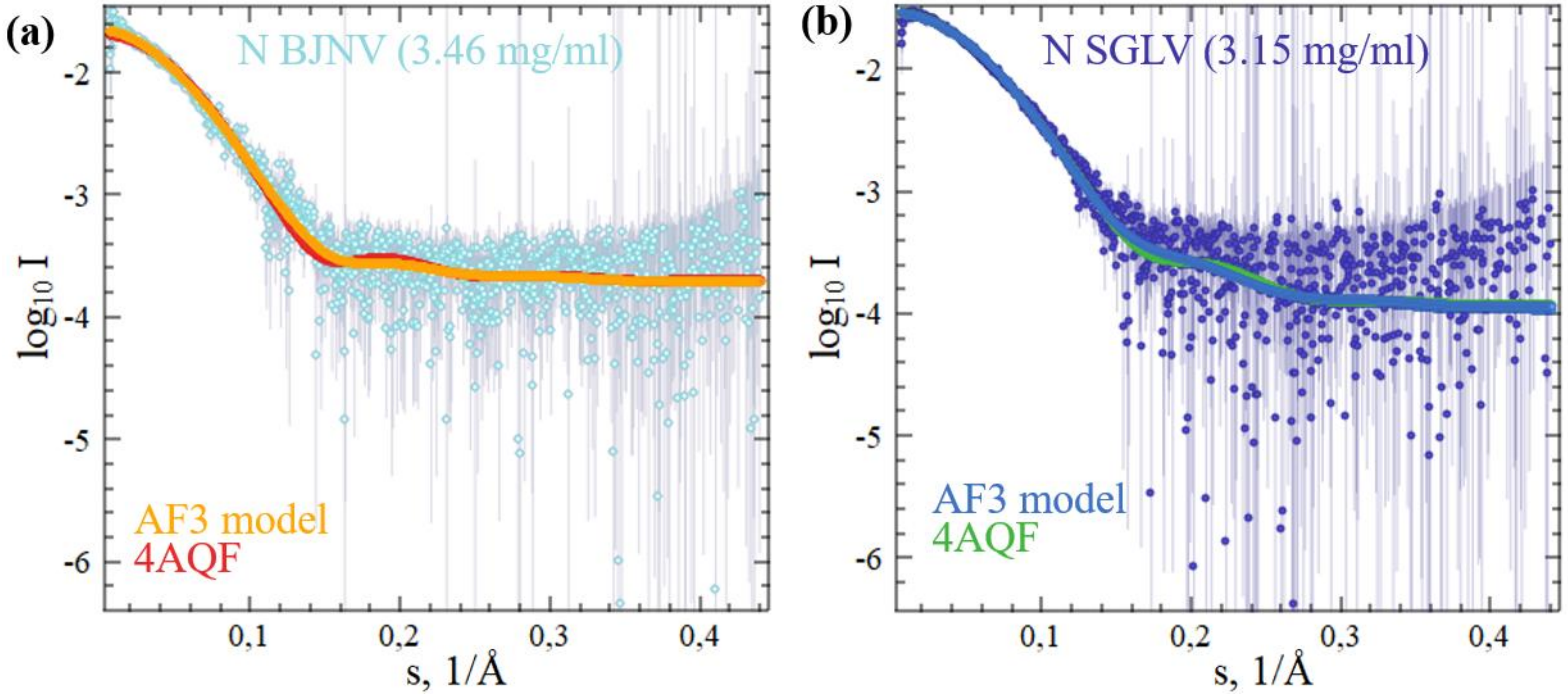

Figure S9. Comparison of experimental SAXS curves with theoretical scattering profiles generated by CRYSOL for BJNV N and SGLV N: **(a)** SAXS curve of BJNV N (blue dots) overlaid with theoretical curves derived from the AlphaFold 3 model (orange) and PDB ID: 4AQF (red); **(b)** SAXS curve of SGLV N (purple dots) compared with theoretical scattering profiles generated from the AlphaFold 3 model (blue) and PDB ID: 4AQF (green).

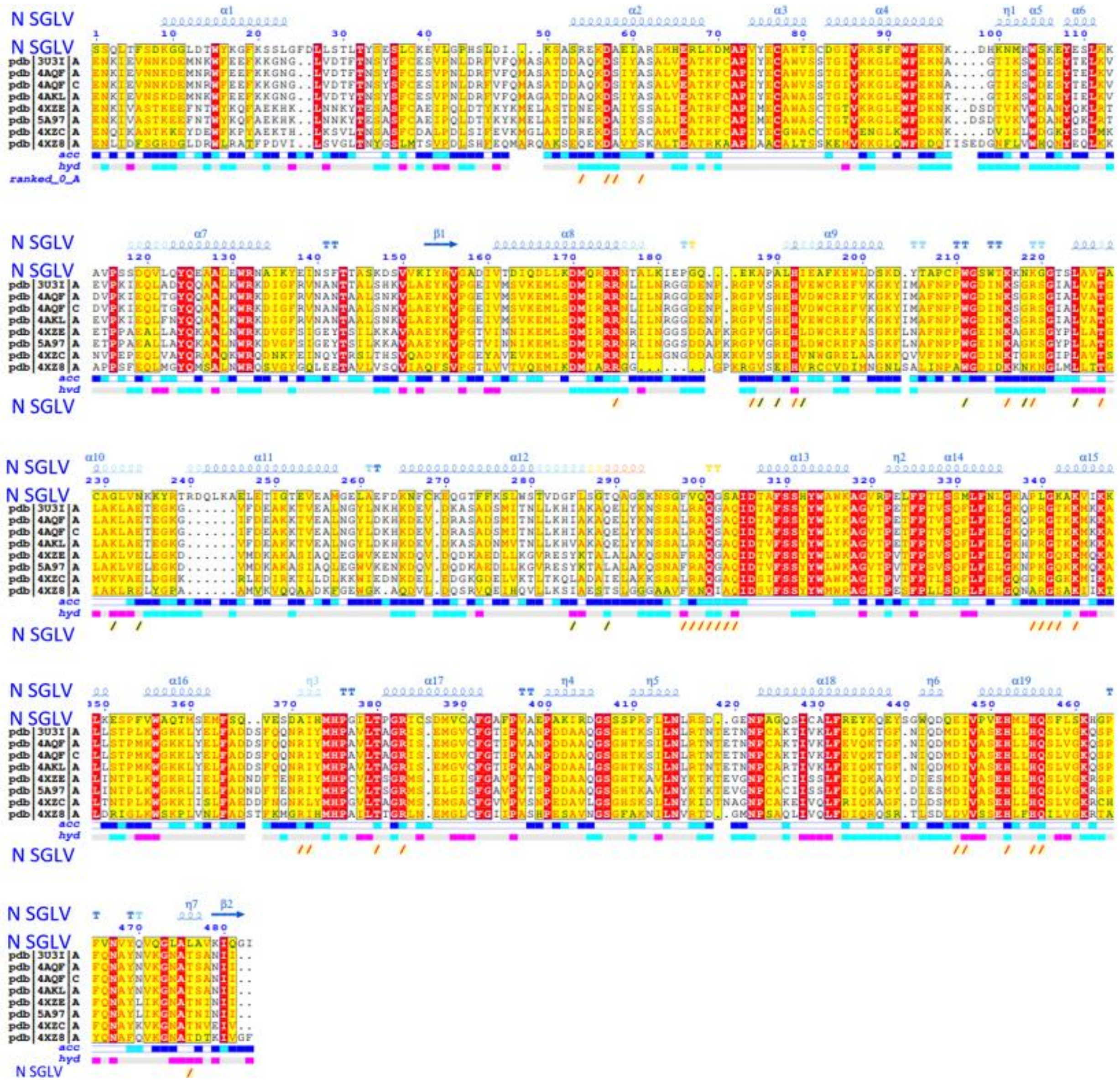

Figure S10. Sequence alignment of the SGLV N with homologous viral structures from PDB. Secondary structures are color-coded according to pLDDT, with blue indicating very high confidence and orange indicating low confidence. Below the structural annotation, the multiple sequence alignment (MSA) highlights residue conservation.

The relative accessibility (labelled 'acc') calculated by DSSP for each residue is shown with a colored bar below the sequences block: white is buried, cyan is intermediate, blue is accessible.The hydropathy (labelled 'hyd') calculated from the query sequence using the Kyte & Doolittle algorithm is shown by a second coloured bar below the accessibility: pink is hydrophobic, grey is intermediate and cyan is hydrophilic. A "/" symbol indicate that the amino acid residue in question has a contact with RNA. A red "/" symbol indicates a contact < 3.2 Å. A black "/" symbol indicates a contact between 3.2 Å and 3.7 Å.

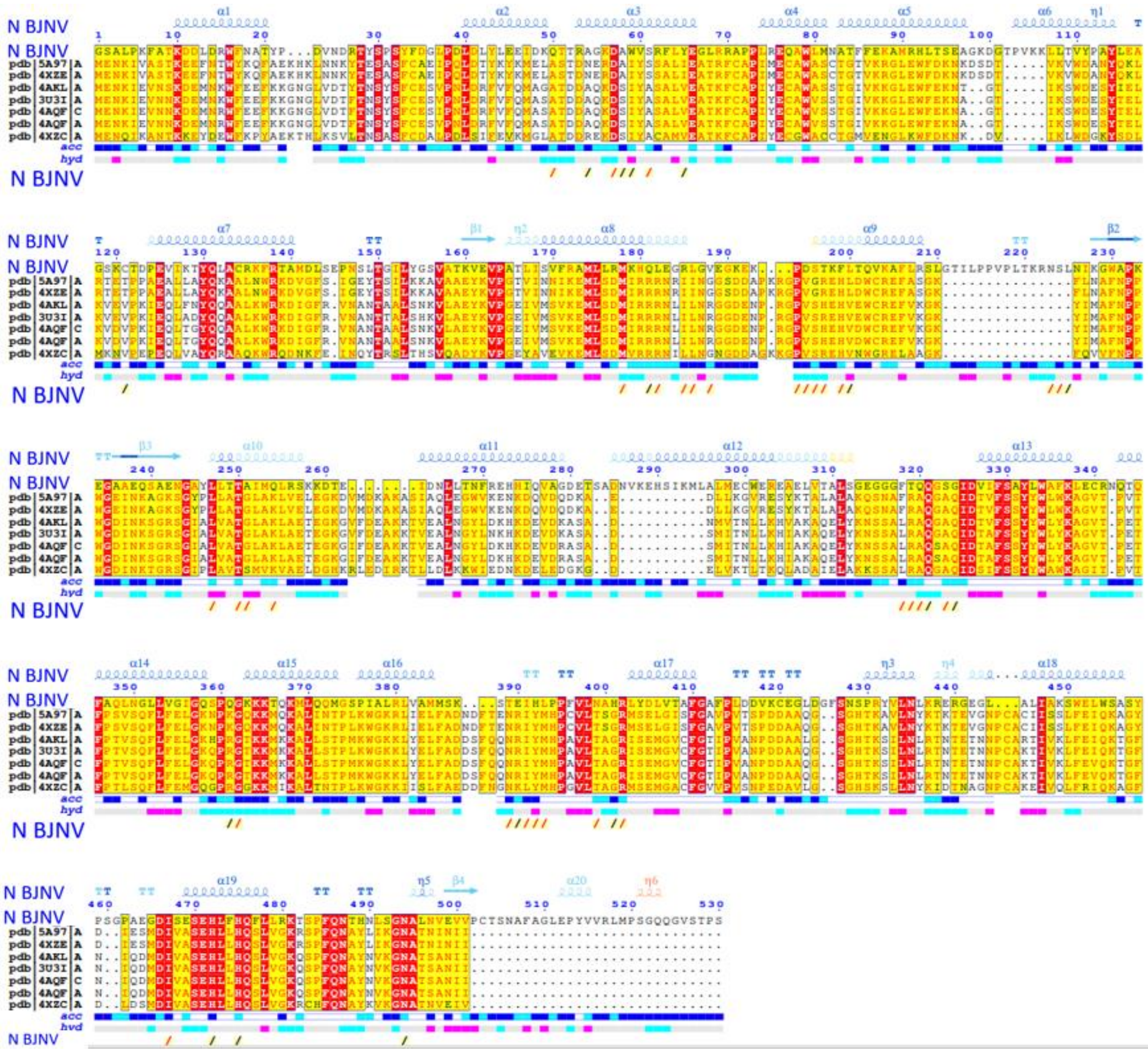

Figure S11. Sequence alignment of the BJNV N with homologous viral structures from PDB. Secondary structures are color-coded according to pLDDT, with blue indicating very high confidence and orange indicating low confidence. Below the structural annotation, MSA highlights residue conservation.

The relative accessibility (labelled 'acc') calculated by DSSP for each residue is shown with a colored bar below the sequences block: white is buried, cyan is intermediate, blue is accessible. The hydropathy (labelled 'hyd') calculated from the query sequence using the Kyte & Doolittle algorithm is shown by a second coloured bar below the accessibility: pink is hydrophobic, grey is intermediate and cyan is hydrophilic. A "/" symbol indicate that the amino acid residue in question has a contact with RNA. A red "/" symbol indicates a contact < 3.2 Å. A black "/" symbol indicates a contact between 3.2 Å and 3.7 Å.

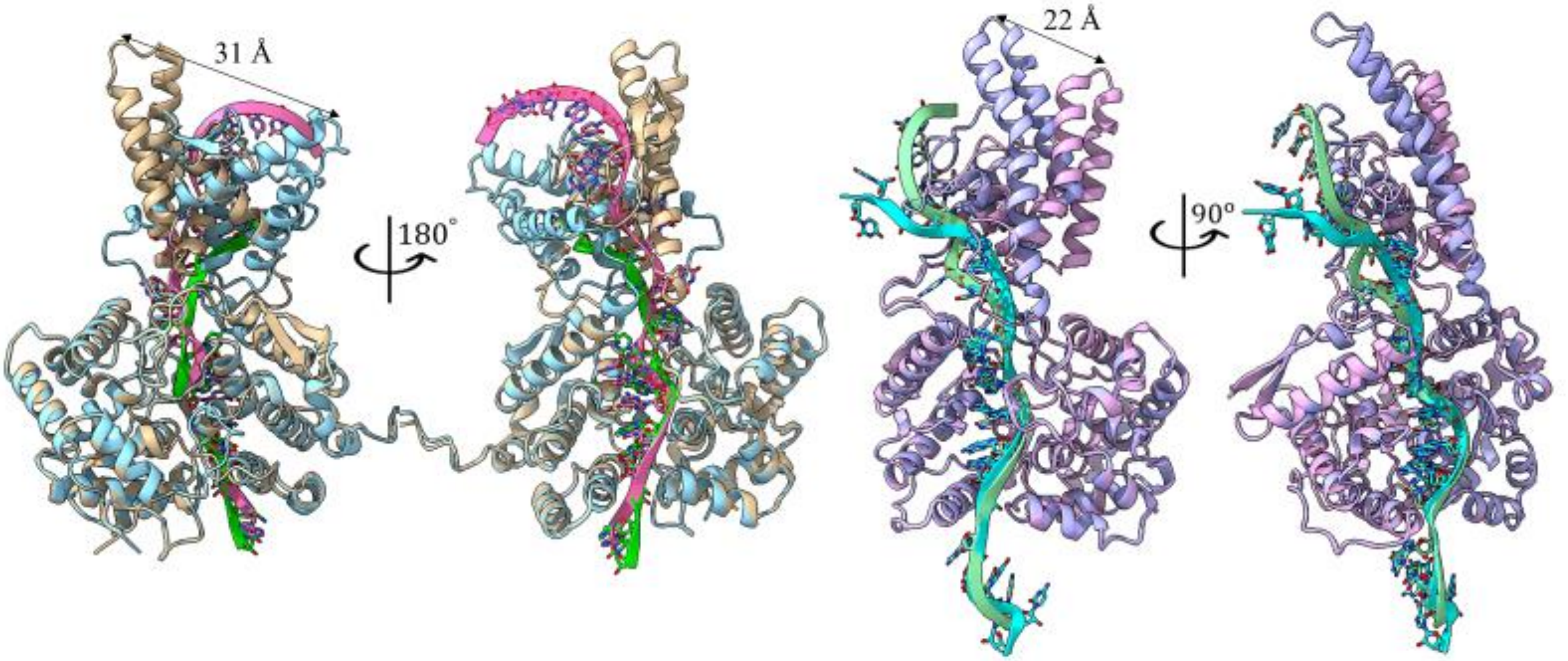

Figure S12. Conformational rearrangements in BJNV N (**left**) and SGLV N (**right**) upon ssRNA(-) binding: **(left)** Alphafold 3 modeling of BJNV N complexed with ssRNA(-) fragments from the BJNV S segment reveals dynamic movement of the stalk domain, resulting in a more compact tertiary structure.; **(right)** Alphafold 3 modeling of SGLV N complexed with ssRNA(-) fragments from the SGLV S segment reveals dynamic movement of the stalk domain, resulting in a more compact tertiary structure.

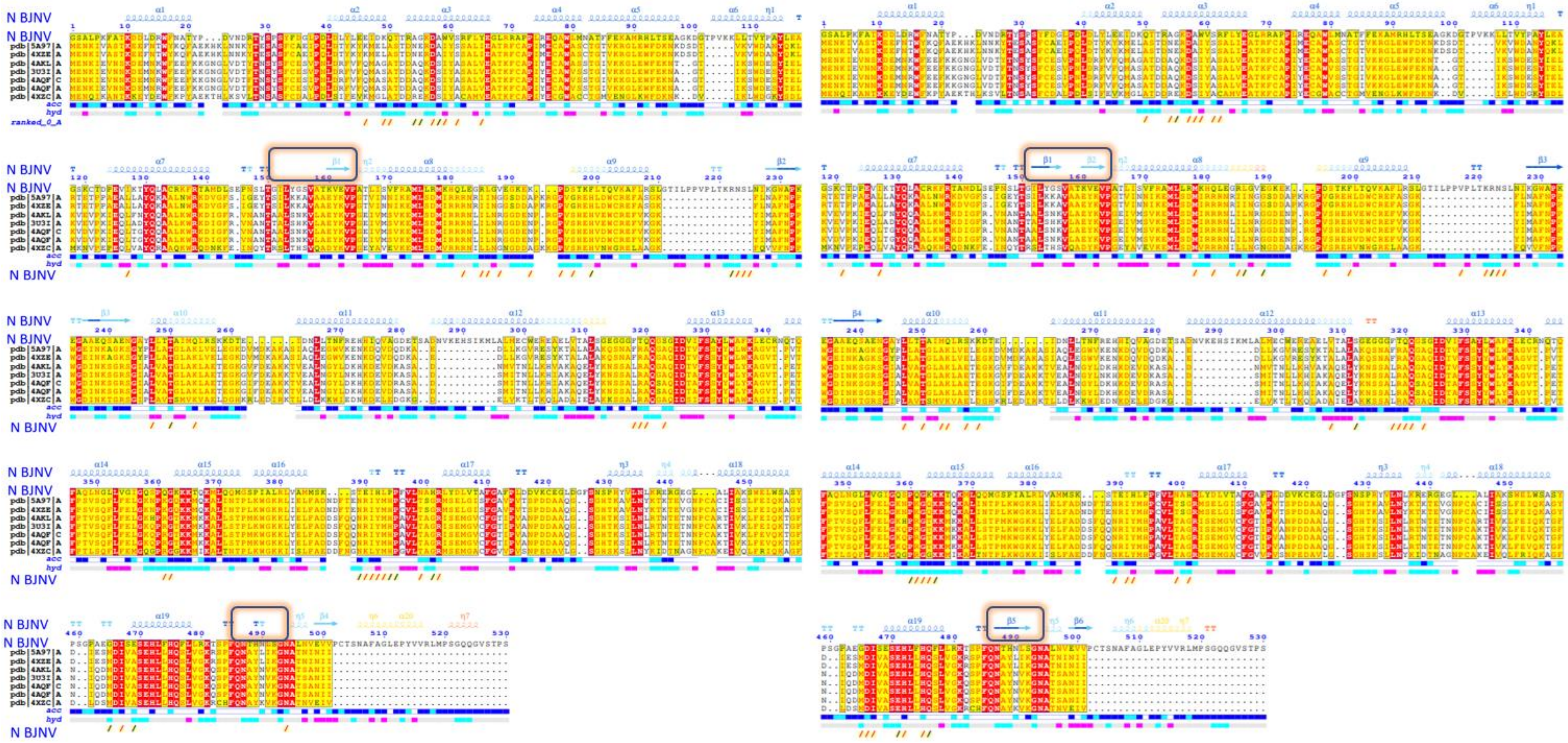

Figure S13. Sequence alignment of structural rearrangements in BJNV N with different ssRNA(-) binding. Alphafold 3 modeling of BJNV N and SGLV N complexes with different ssRNA(-) fragments reveals structural rearrangements, highlighted by boxes in the figure.

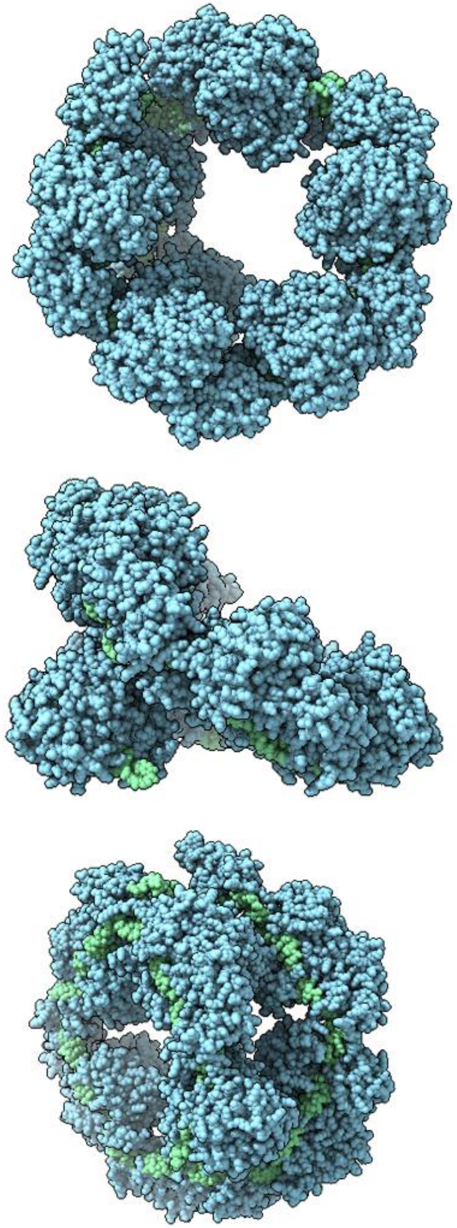

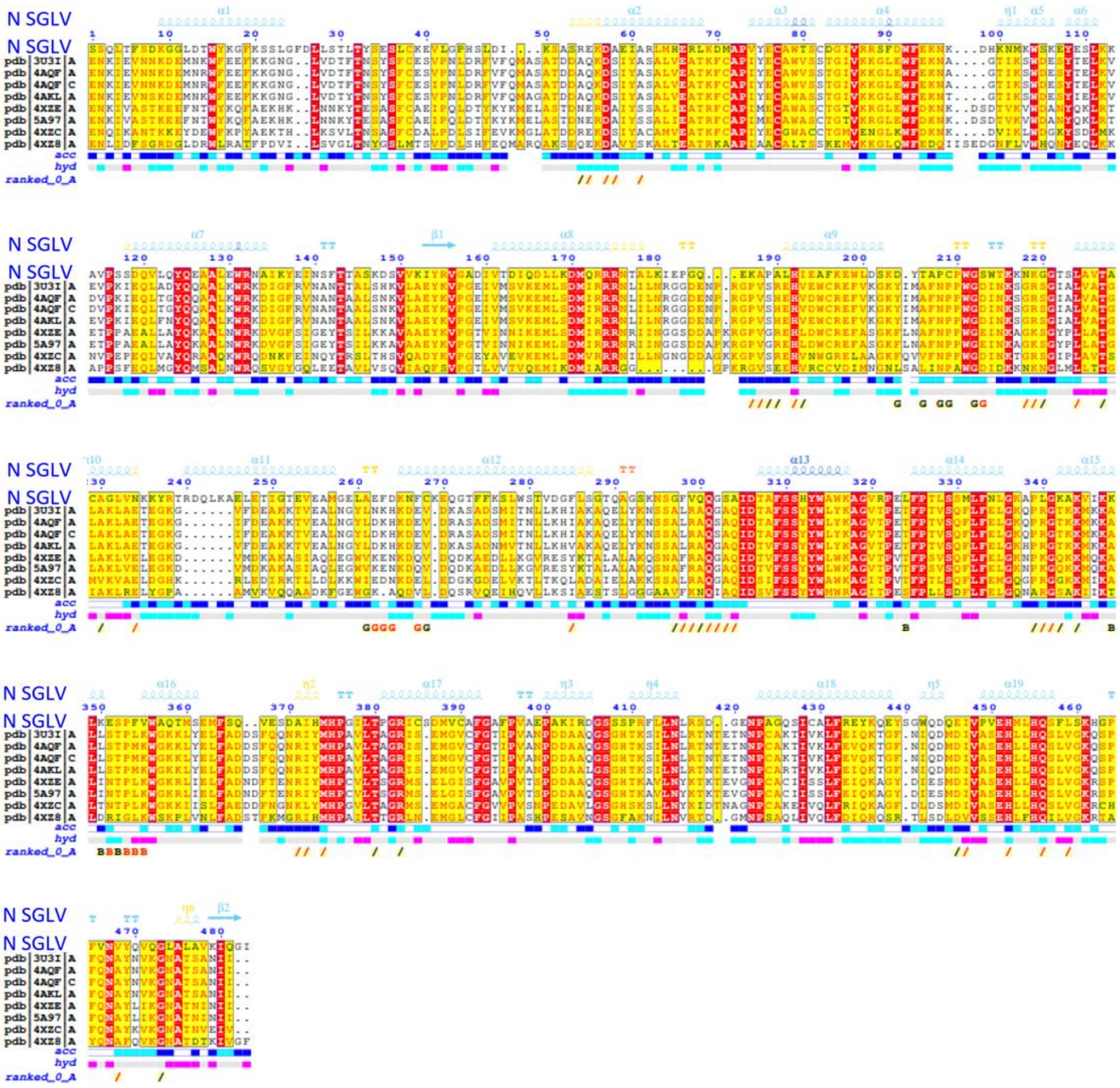

N SGLV
pdb 3U3I A
pdb 4AQF A
pdb 4AQF C
pdb 4AKL A
pdb 4XZE A
pdb 5A97 A
pdb 4XZC A
pdb 4XZ8 A
acc
hyd
ranked_0_A

Figure S14. AlphaFold 3 structural models of SGLV RNP heptamer complexes (top and side views) (**left**). In SGLV RNPs, N monomers are shown in blue, and the RNA is colored green. Sequence alignment of the SGLV N heptamer with homologous viral structures from PDB (**right**). Secondary structures are color-coded according to pLDDT, with blue indicating very high confidence and orange indicating low confidence. Below the structural annotation, MSA highlights residue conservation.

The relative accessibility (labelled 'acc') calculated by DSSP for each residue is shown with a colored bar below the sequences block: white is buried, cyan is intermediate, blue is accessible. The hydropathy (labelled 'hyd') calculated from the query sequence using the Kyte & Doolittle algorithm is shown by a second coloured bar below the accessibility: pink is hydrophobic, grey is intermediate and cyan is hydrophilic. A "/" symbol indicate that the amino acid residue in question has a contact with RNA. A red "/" symbol indicates a contact < 3.2 Å. A black "/" symbol indicates a contact between 3.2 Å and 3.7 Å.

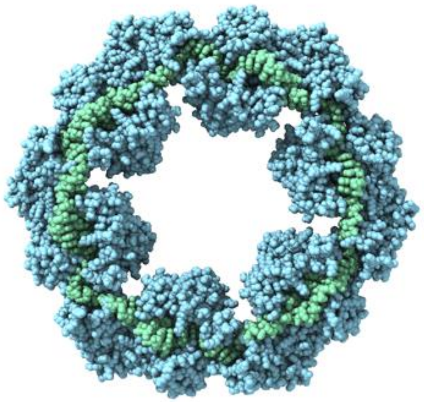

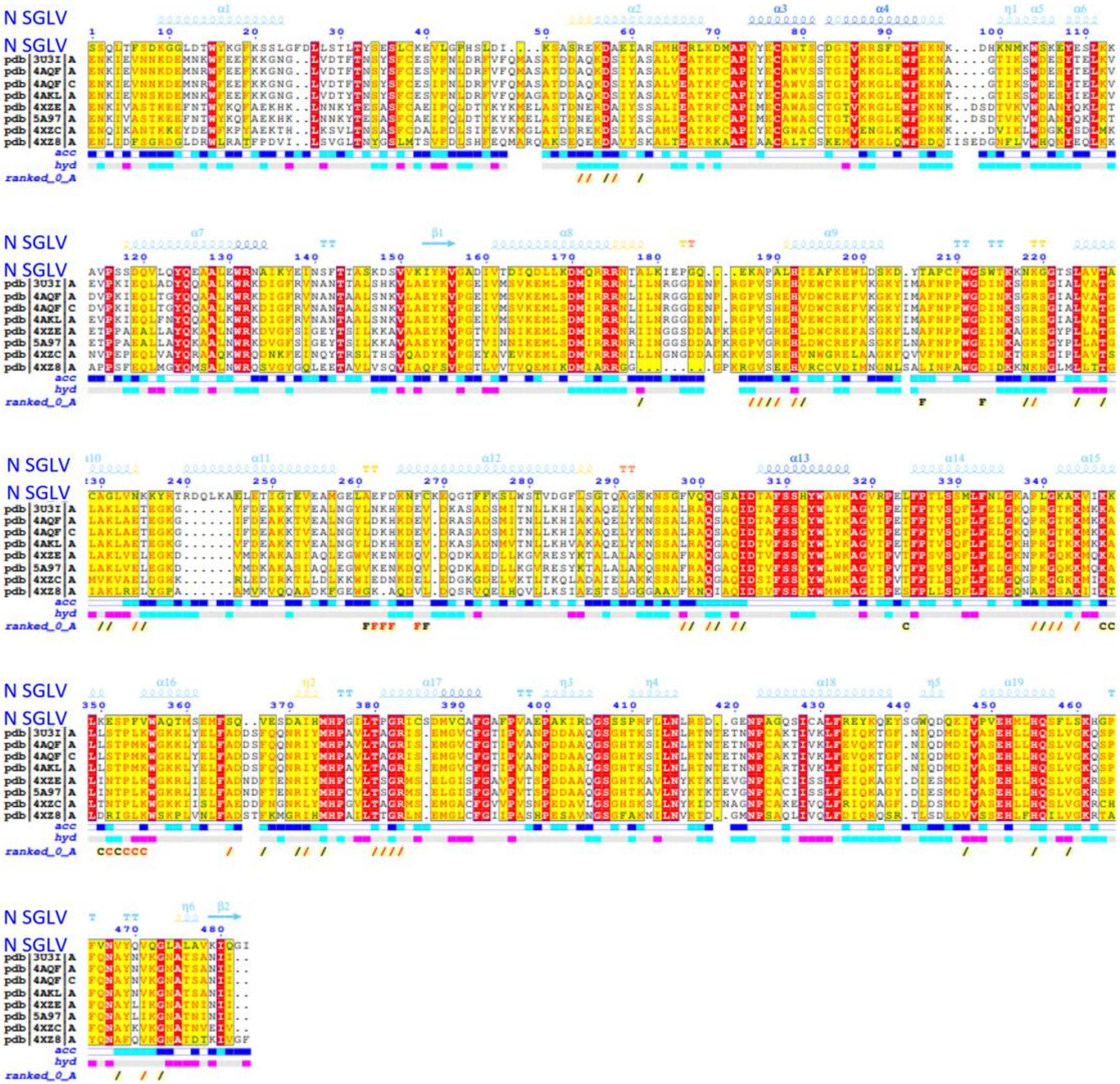
N SGLV
pdb|3U3I|A
pdb|4AQF|A
pdb|4AQF|C
pdb|4AKL|A
pdb|4XZE|A
pdb|5A97|A
pdb|4XZC|A
pdb|4XZ8|A
acc
hyd
ranked_0_A

Figure S15. AlphaFold 3 structural models of SGLV RNP hexamer complexes (top view) (**left**). In SGLV RNPs, N monomers are shown in blue, and the RNA is colored green. Sequence alignment of the SGLV N hexamer with homologous viral structures from PDB (**right**). Secondary structures are color-coded according to pLDDT, with blue indicating very high confidence and orange indicating low confidence. Below the structural annotation, MSA highlights residue conservation.

The relative accessibility (labelled 'acc') calculated by DSSP for each residue is shown with a colored bar below the sequences block: white is buried, cyan is intermediate, blue is accessible. The hydropathy (labelled 'hyd') calculated from the query sequence using the Kyte & Doolittle algorithm is shown by a second coloured bar below the accessibility: pink is hydrophobic, grey is intermediate and cyan is hydrophilic. A "/" symbol indicate that the amino acid residue in question has a contact with RNA. A red "/" symbol indicates a contact < 3.2 Å. A black "/" symbol indicates a contact between 3.2 Å and 3.7 Å.

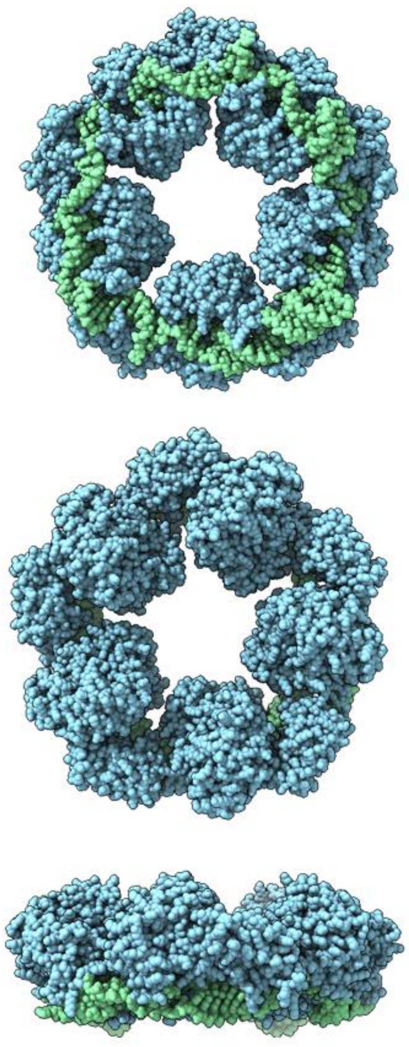

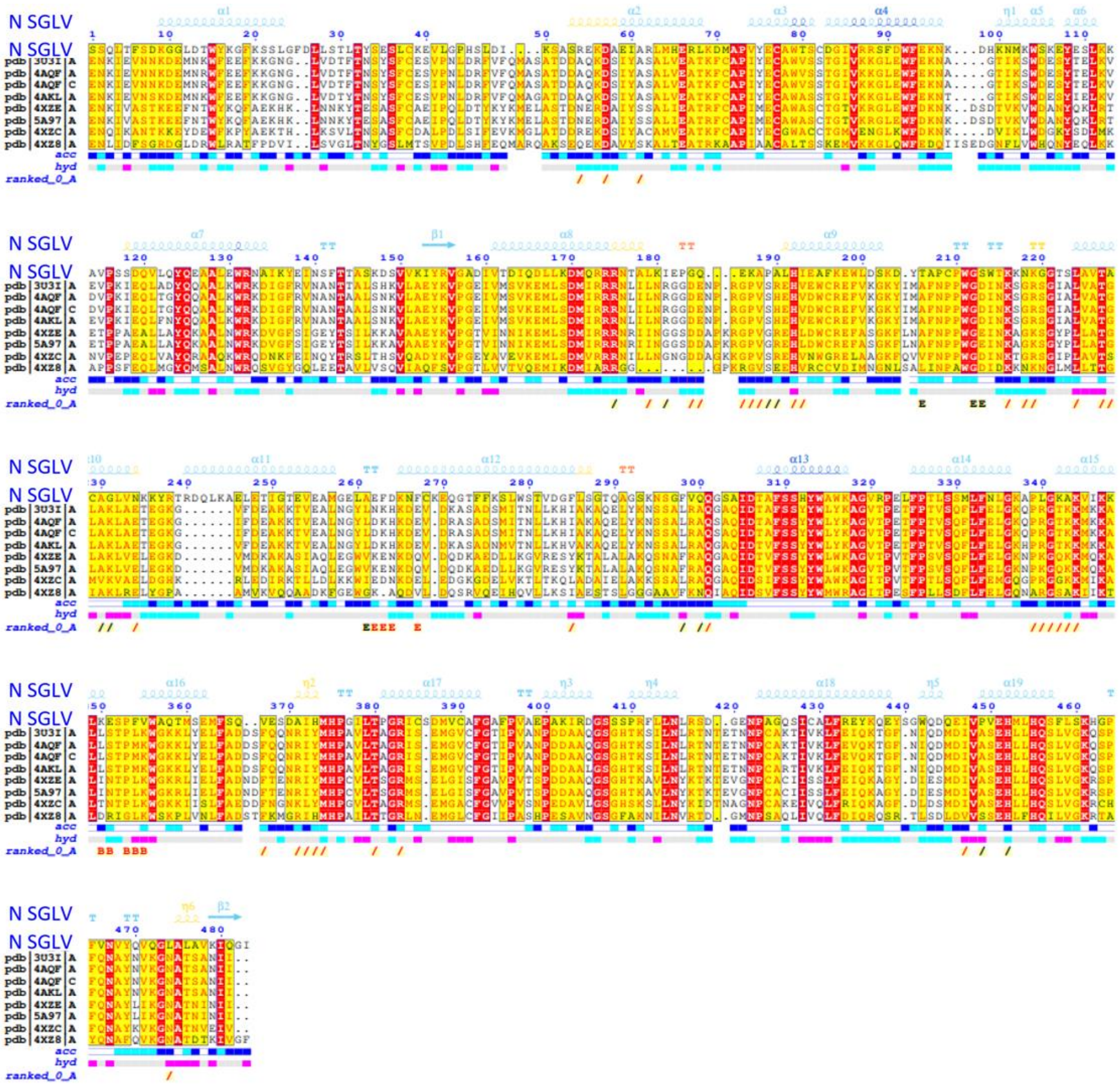
N SGLV
N SGLV
pdb|3U3I|A
pdb|4AQF|A
pdb|4AQF|C
pdb|4AKL|A
pdb|4XZE|A
pdb|5A97|A
pdb|4XZC|A
pdb|4XZ8|A
acc
hyd
ranked_0_A

Figure S16. AlphaFold 3 structural models of SGLV RNP pentamer complexes (top, bottom and side views) (**left**). In SGLV RNPs, N monomers are shown in blue, and the RNA is colored green. Sequence alignment of the SGLV N pentamer with homologous viral structures from PDB (**right**). Secondary structures are color-coded according to pLDDT, with blue indicating very high confidence and orange indicating low confidence. Below the structural annotation, MSA highlights residue conservation.

The relative accessibility (labelled 'acc') calculated by DSSP for each residue is shown with a colored bar below the sequences block: white is buried, cyan is intermediate, blue is accessible. The hydropathy (labelled 'hyd') calculated from the query sequence using the Kyte & Doolittle algorithm is shown by a second coloured bar below the accessibility: pink is hydrophobic, grey is intermediate and cyan is hydrophilic. A "/" symbol indicate that the amino acid residue in question has a contact with RNA. A red "/" symbol indicates a contact < 3.2 Å. A black "/" symbol indicates a contact between 3.2 Å and 3.7 Å.

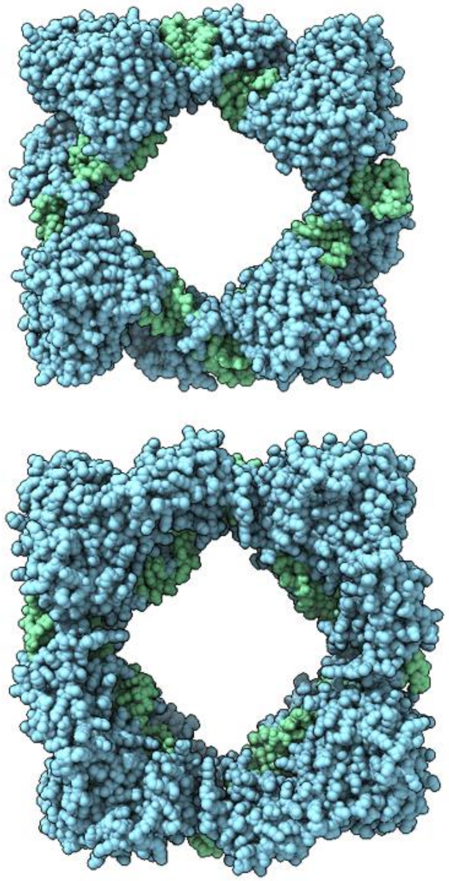

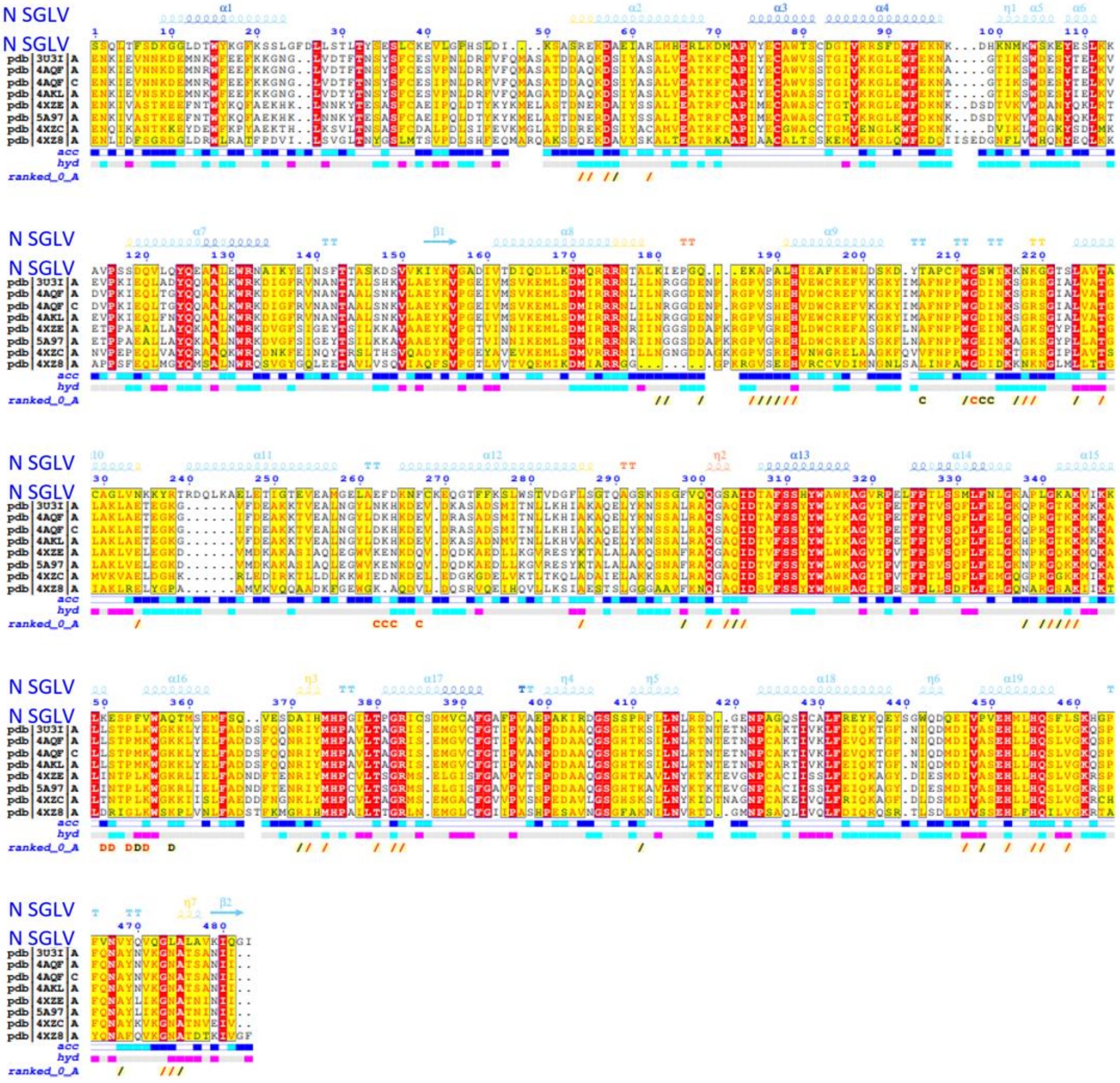
N SGLV
pdb|3U3I|A
pdb|4AQF|A
pdb|4AQF|C
pdb|4AKL|A
pdb|4XZE|A
pdb|5A97|A
pdb|4XZC|A
pdb|4XZ8|A
acc
hyd
ranked_0_A

Figure S17. AlphaFold 3 structural models of SGLV RNP tetramer complexes (top and bottom views) (**left**). In SGLV RNPs, N monomers are shown in blue, and the RNA is colored green. Sequence alignment of the SGLV N tetramer with homologous viral structures from PDB (**right**). Secondary structures are color-coded according to pLDDT, with blue indicating very high confidence and orange indicating low confidence. Below the structural annotation, MSA highlights residue conservation.

The relative accessibility (labelled 'acc') calculated by DSSP for each residue is shown with a colored bar below the sequences block: white is buried, cyan is intermediate, blue is accessible. The hydropathy (labelled 'hyd') calculated from the query sequence using the Kyte & Doolittle algorithm is shown by a second coloured bar below the accessibility: pink is hydrophobic, grey is intermediate and cyan is hydrophilic. A "/" symbol indicate that the amino acid residue in question has a contact with RNA. A red "/" symbol indicates a contact < 3.2 Å. A black "/" symbol indicates a contact between 3.2 Å and 3.7 Å.

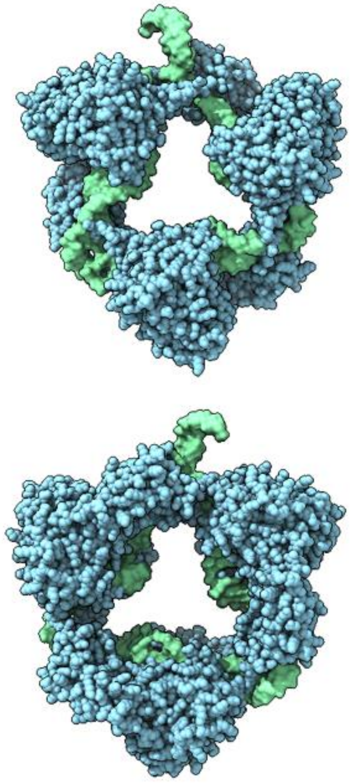

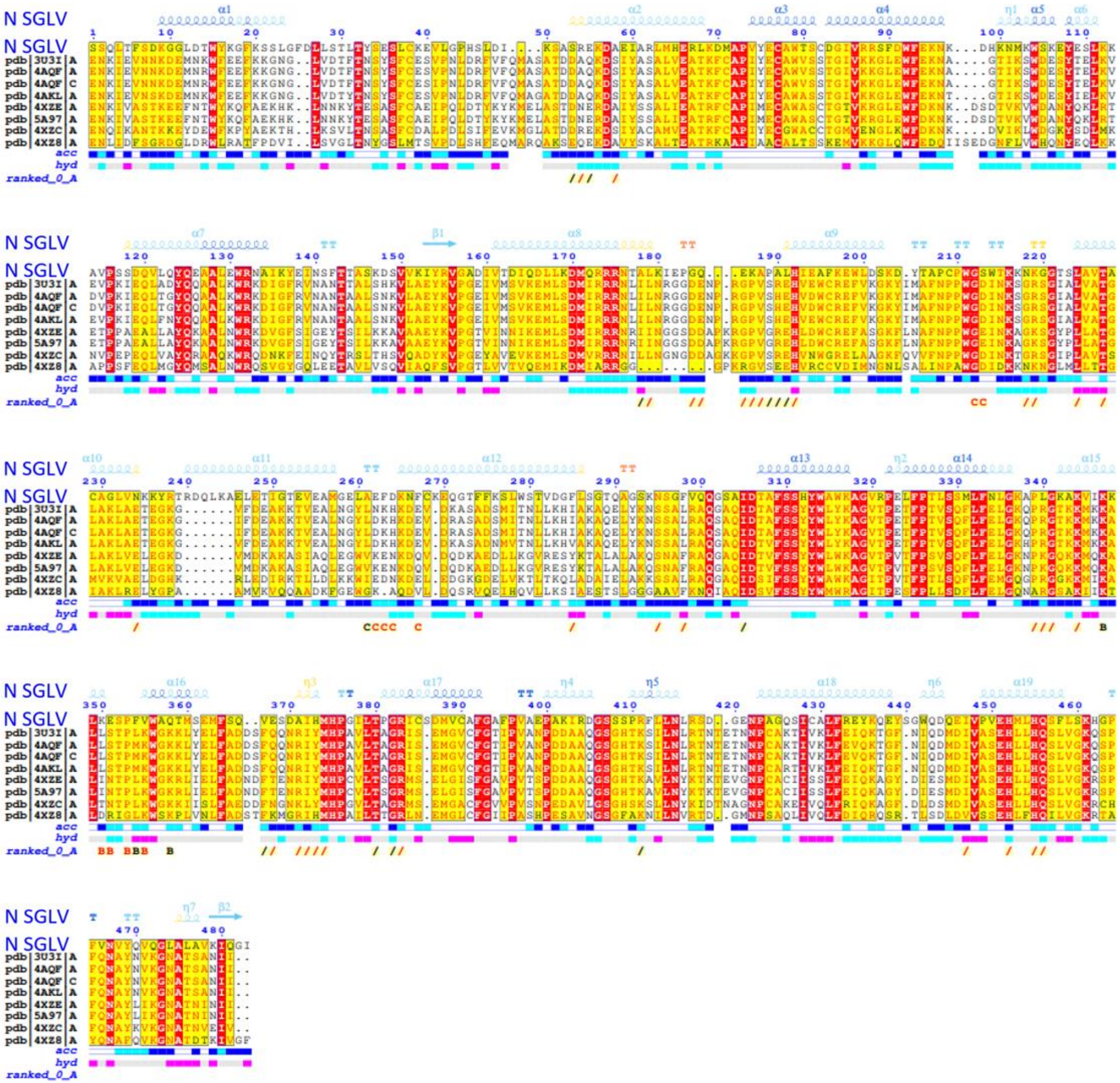
N SGLV
pdb|3U3I|A
pdb|4AQF|A
pdb|4AQF|C
pdb|4AKL|A
pdb|4XZE|A
pdb|5A97|A
pdb|4XZC|A
pdb|4XZ8|A
acc
hyd
ranked_0_A

Figure S18. AlphaFold 3 structural models of SGLV RNP trimer complexes (top and bottom views) (**left**). In SGLV RNPs, N monomers are shown in blue, and the RNA is colored green. Sequence alignment of the SGLV N trimer with homologous viral structures from PDB (**right**). Secondary structures are color-coded according to pLDDT, with blue indicating very high confidence and orange indicating low confidence. Below the structural annotation, MSA highlights residue conservation.

The relative accessibility (labelled 'acc') calculated by DSSP for each residue is shown with a colored bar below the sequences block: white is buried, cyan is intermediate, blue is accessible. The hydropathy (labelled 'hyd') calculated from the query sequence using the Kyte & Doolittle algorithm is shown by a second coloured bar below the accessibility: pink is hydrophobic, grey is intermediate and cyan is hydrophilic. A "/" symbol indicate that the amino acid residue in question has a contact with RNA. A red "/" symbol indicates a contact < 3.2 Å. A black "/" symbol indicates a contact between 3.2 Å and 3.7 Å.

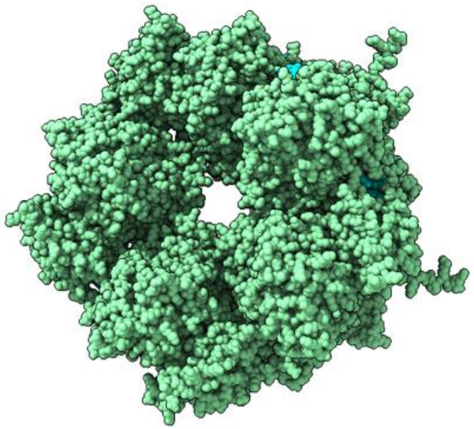

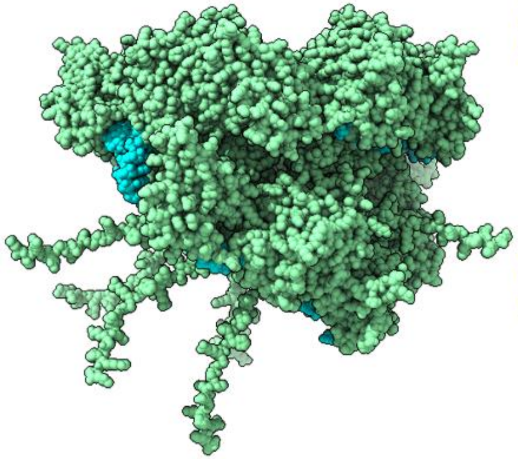

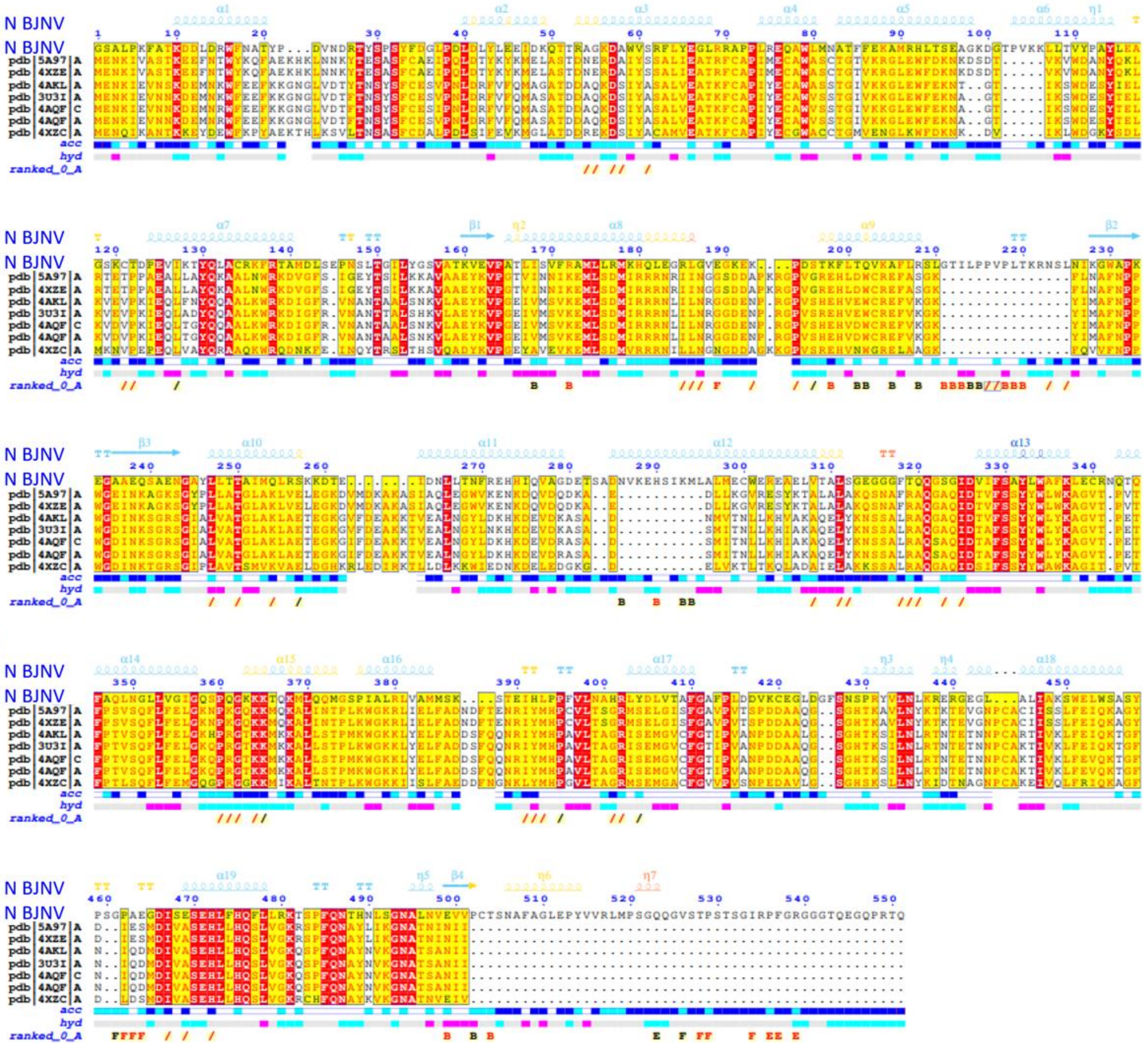
N BJNV
pdb|5A97|A
pdb|4XZE|A
pdb|4AKL|A
pdb|3U3I|A
pdb|4AQF|C
pdb|4AQF|A
pdb|4XZC|A
acc
hyd
ranked_0_A

Figure S19. AlphaFold 3 structural models of BJNV RNP heptamer complexes (top and side views) (**left**). In BJNV RNPs, N monomers are shown in green, and the RNA is colored blue. Sequence alignment of the BJNV N heptamer with homologous viral structures from PDB (**right**). Secondary structures are color-coded according to pLDDT, with blue indicating very high confidence and orange indicating low confidence. Below the structural annotation, MSA highlights residue conservation.

The relative accessibility (labelled 'acc') calculated by DSSP for each residue is shown with a colored bar below the sequences block: white is buried, cyan is intermediate, blue is accessible. The hydropathy (labelled 'hyd') calculated from the query sequence using the Kyte & Doolittle algorithm is shown by a second coloured bar below the accessibility: pink is hydrophobic, grey is intermediate and cyan is hydrophilic. A "/" symbol indicate that the amino acid residue in question has a contact with RNA. A red "/" symbol indicates a contact < 3.2 Å. A black "/" symbol indicates a contact between 3.2 Å and 3.7 Å.

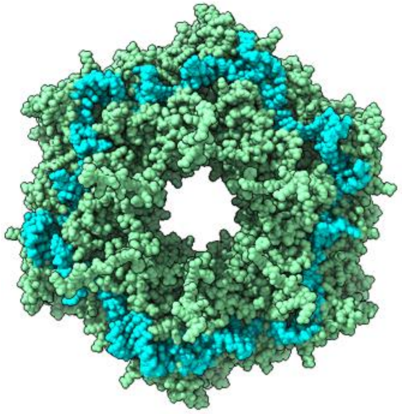

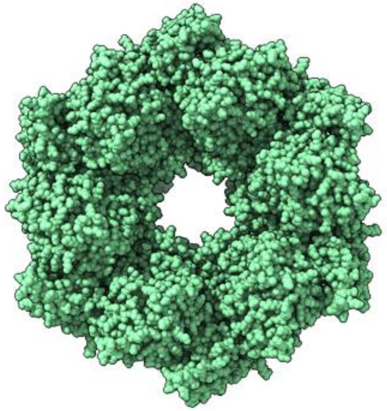

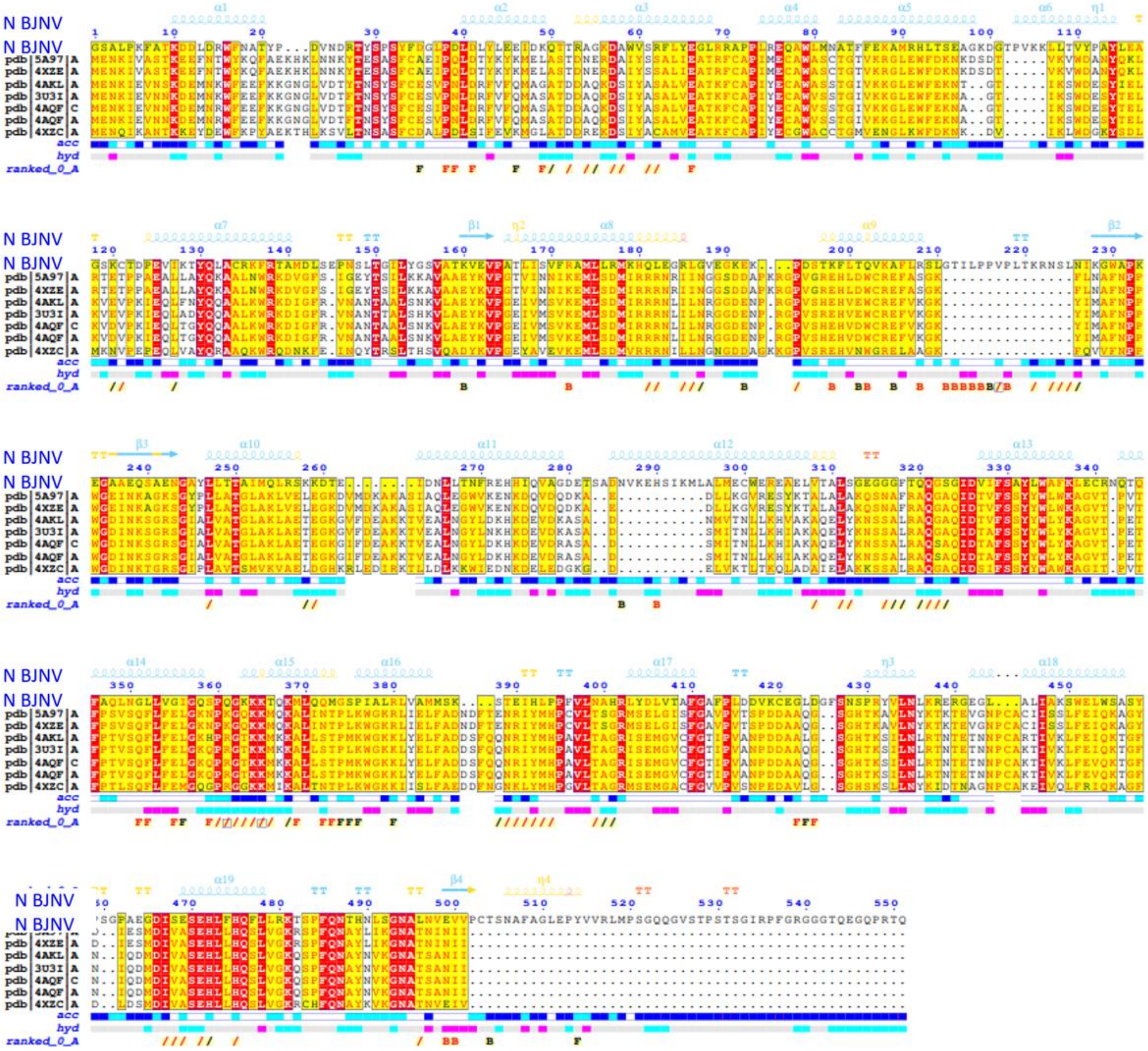
N BJNV
pdb|5A97|A
pdb|4XZE|A
pdb|4AKL|A
pdb|3U3I|A
pdb|4AQF|C
pdb|4AQF|A
pdb|4XZC|A
acc
hyd
ranked_0_A

Figure S20. AlphaFold 3 structural models of BJNV RNP hexamer complexes (top and bottom views) (**left**). In BJNV RNPs, N monomers are shown in green, and the RNA is colored blue. Sequence alignment of the BJNV N hexamer with homologous viral structures from PDB (**right**). Secondary structures are color-coded according to pLDDT, with blue indicating very high confidence and orange indicating low confidence. Below the structural annotation, MSA highlights residue conservation.

The relative accessibility (labelled 'acc') calculated by DSSP for each residue is shown with a colored bar below the sequences block: white is buried, cyan is intermediate, blue is accessible. The hydropathy (labelled 'hyd') calculated from the query sequence using the Kyte & Doolittle algorithm is shown by a second coloured bar below the accessibility: pink is hydrophobic, grey is intermediate and cyan is hydrophilic. A "/" symbol indicate that the amino acid residue in question has a contact with RNA. A red "/" symbol indicates a contact < 3.2 Å. A black "/" symbol indicates a contact between 3.2 Å and 3.7 Å.

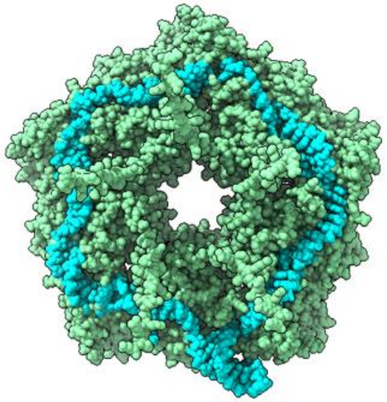

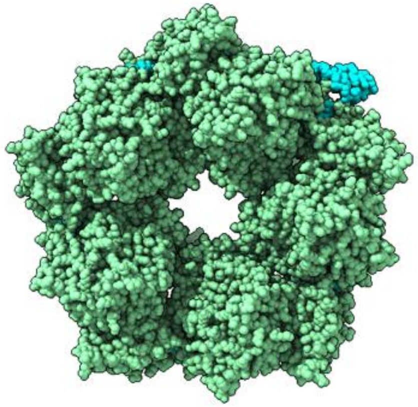

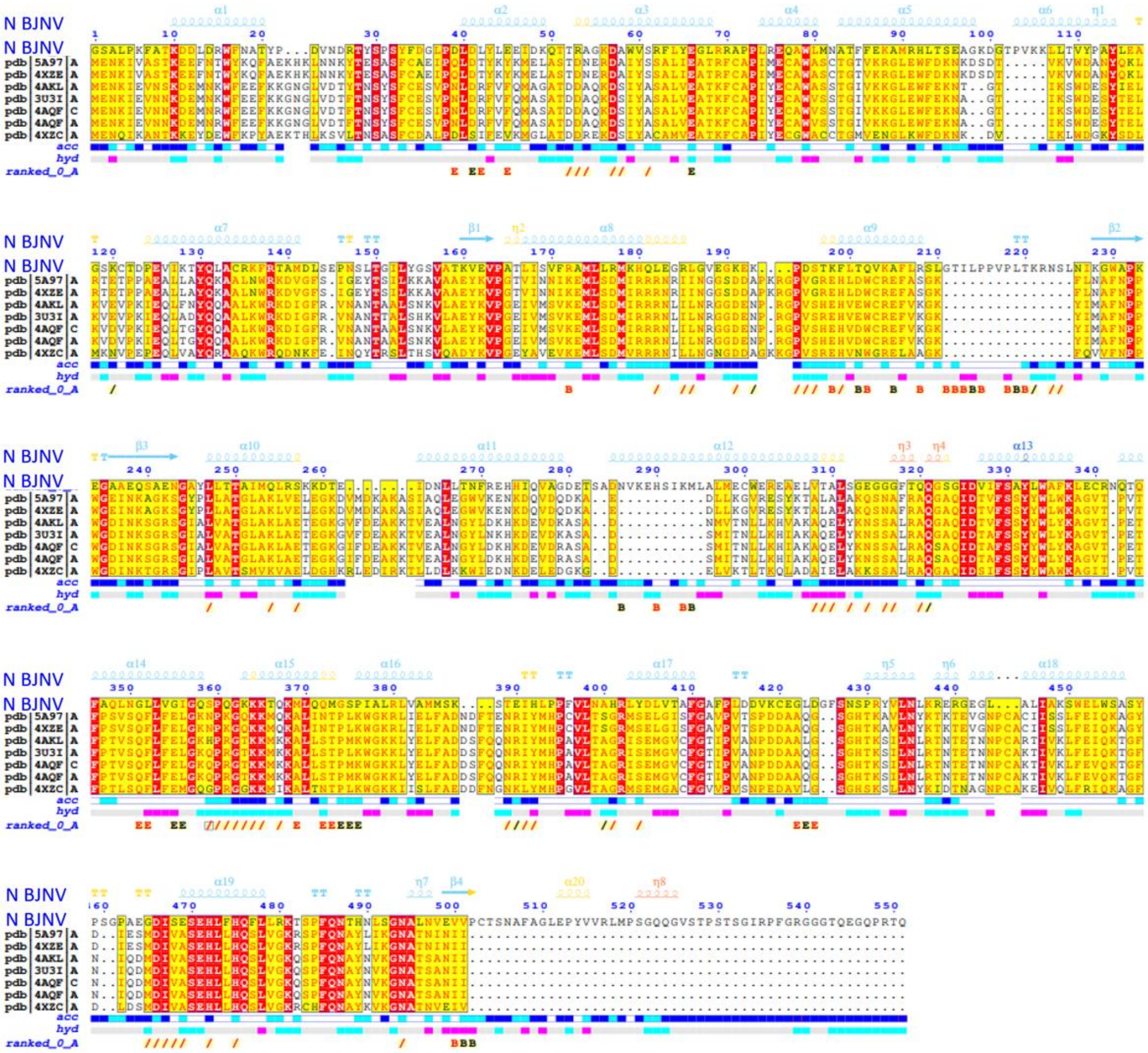
N BJNV
pdb|5A97|A
pdb|4XZE|A
pdb|4AKL|A
pdb|3U3I|A
pdb|4AQF|C
pdb|4AQF|A
pdb|4XZC|A
acc
hyd
ranked_0_A

Figure S21. AlphaFold 3 structural models of BJNV RNP pentamer complexes (top and bottom views) (**left**). In BJNV RNPs, N monomers are shown in green, and the RNA is colored blue. Sequence alignment of the BJNV N pentamer with homologous viral structures from PDB (**right**). Secondary structures are color-coded according to pLDDT, with blue indicating very high confidence and orange indicating low confidence. Below the structural annotation, MSA highlights residue conservation.

The relative accessibility (labelled 'acc') calculated by DSSP for each residue is shown with a colored bar below the sequences block: white is buried, cyan is intermediate, blue is accessible. The hydropathy (labelled 'hyd') calculated from the query sequence using the Kyte & Doolittle algorithm is shown by a second coloured bar below the accessibility: pink is hydrophobic, grey is intermediate and cyan is hydrophilic. A "/" symbol indicate that the amino acid residue in question has a contact with RNA. A red "/" symbol indicates a contact < 3.2 Å. A black "/" symbol indicates a contact between 3.2 Å and 3.7 Å.

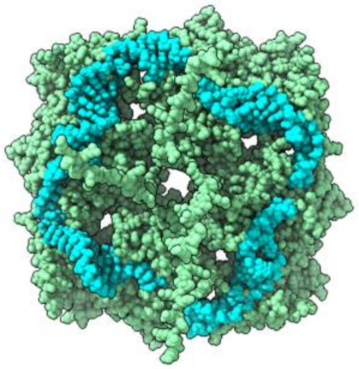

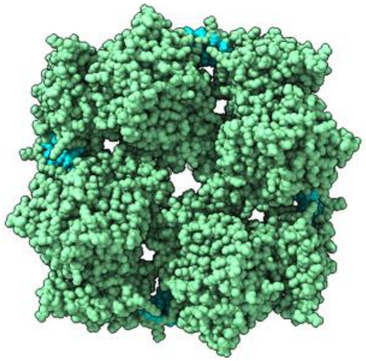

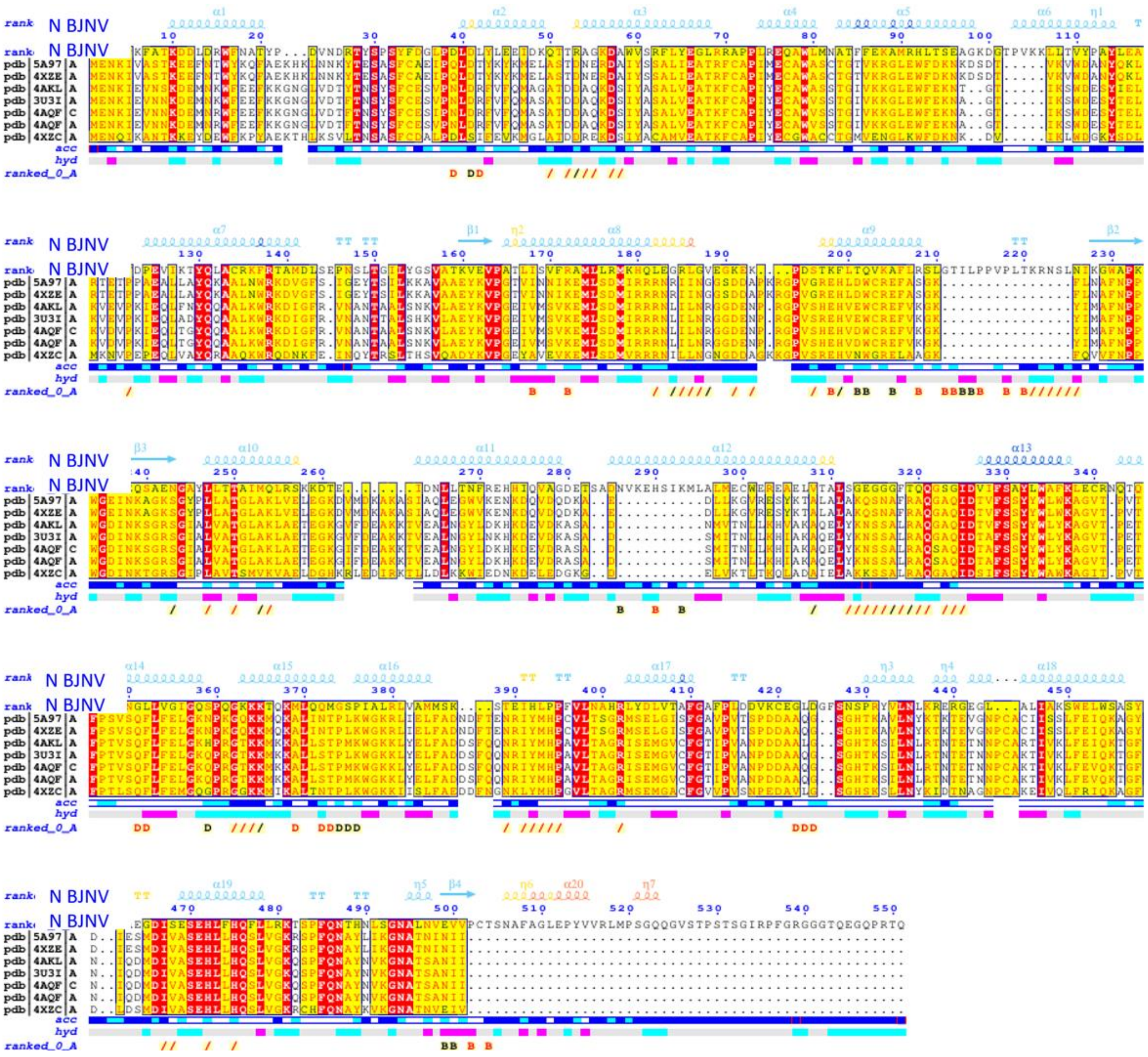
rank
N BJNV
pdb|5A97|A
pdb|4XZE|A
pdb|4AKL|A
pdb|3U3I|A
pdb|4AQF|C
pdb|4AQF|A
pdb|4XZC|A
acc
hyd
ranked_0_A

Figure S22. AlphaFold 3 structural models of BJNV RNP tetramer complexes (top and bottom views) (**left**). In BJNV RNPs, N monomers are shown in green, and the RNA is colored blue. Sequence alignment of the BJNV N tetramer with homologous viral structures from PDB (**right**). Secondary structures are color-coded according to pLDDT, with blue indicating very high confidence and orange indicating low confidence. Below the structural annotation, MSA highlights residue conservation.

The relative accessibility (labelled 'acc') calculated by DSSP for each residue is shown with a colored bar below the sequences block: white is buried, cyan is intermediate, blue is accessible. The hydropathy (labelled 'hyd') calculated from the query sequence using the Kyte & Doolittle algorithm is shown by a second coloured bar below the accessibility: pink is hydrophobic, grey is intermediate and cyan is hydrophilic. A "/" symbol indicate that the amino acid residue in question has a contact with RNA. A red "/" symbol indicates a contact < 3.2 Å. A black "/" symbol indicates a contact between 3.2 Å and 3.7 Å.

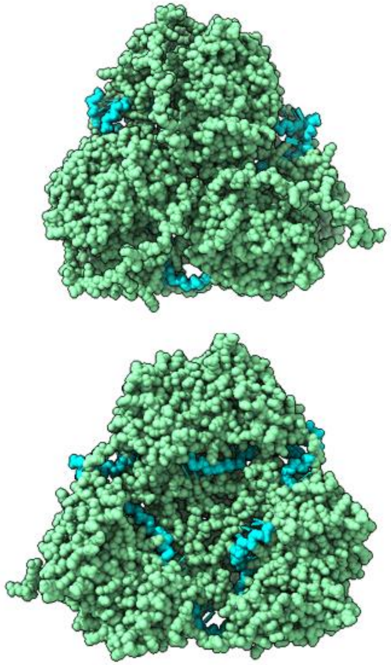

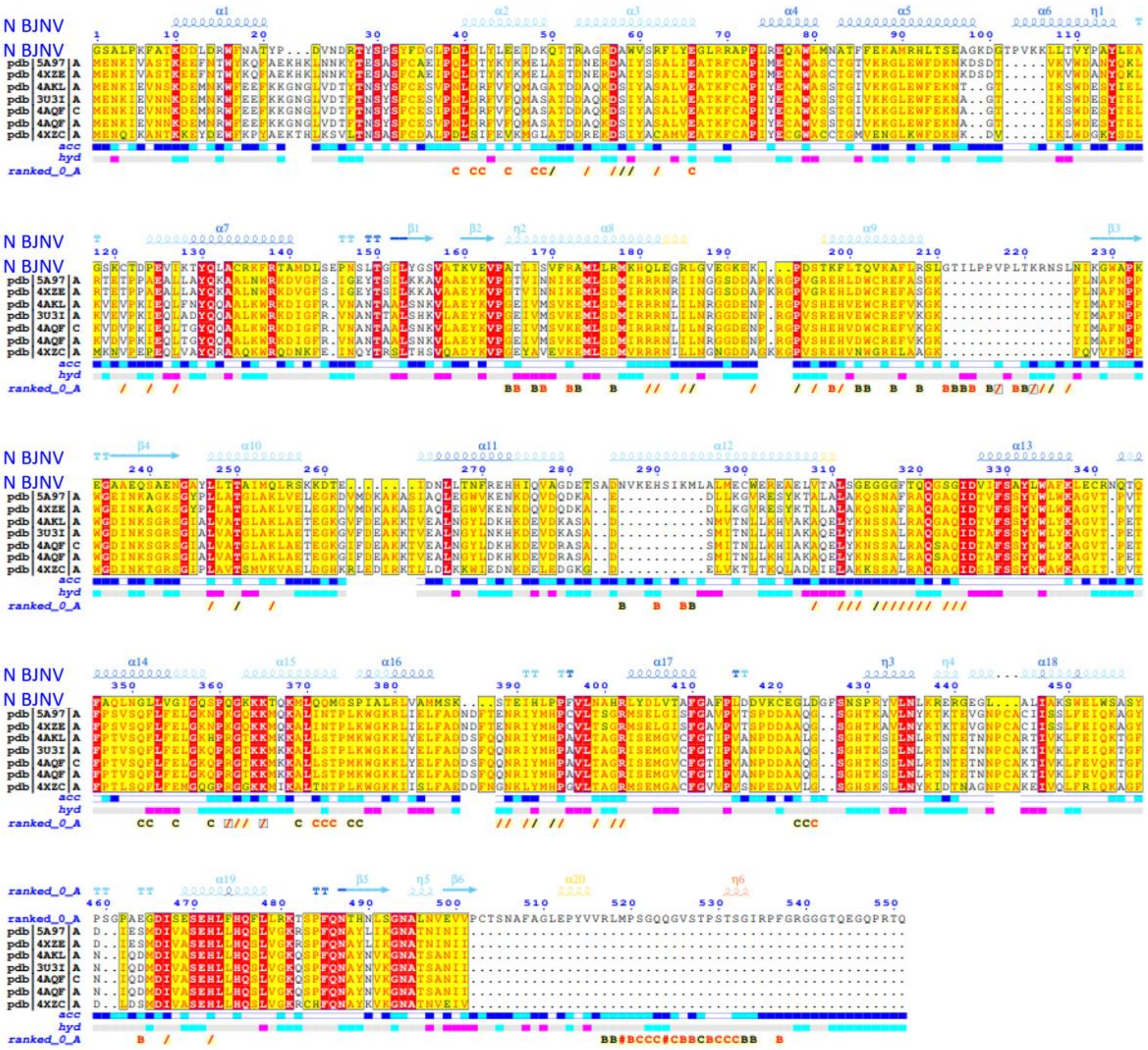
N BJNV
N BJNV
pdb|5A97|A
pdb|4XZE|A
pdb|4AKL|A
pdb|3U3I|A
pdb|4AQF|C
pdb|4AQF|A
pdb|4XZC|A
acc
hyd
ranked_0_A

Figure S23. AlphaFold 3 structural models of BJNV RNP trimer complexes (top and bottom views) (**left**). In BJNV RNPs, N monomers are shown in green, and the RNA is colored blue. Sequence alignment of the BJNV N trimer with homologous viral structures from PDB (**right**). Secondary structures are color-coded according to pLDDT, with blue indicating very high confidence and orange indicating low confidence. Below the structural annotation, MSA highlights residue conservation.

The relative accessibility (labelled 'acc') calculated by DSSP for each residue is shown with a colored bar below the sequences block: white is buried, cyan is intermediate, blue is accessible. The hydropathy (labelled 'hyd') calculated from the query sequence using the Kyte & Doolittle algorithm is shown by a second coloured bar below the accessibility: pink is hydrophobic, grey is intermediate and cyan is hydrophilic. A "/" symbol indicate that the amino acid residue in question has a contact with RNA. A red "/" symbol indicates a contact < 3.2 Å. A black "/" symbol indicates a contact between 3.2 Å and 3.7 Å.

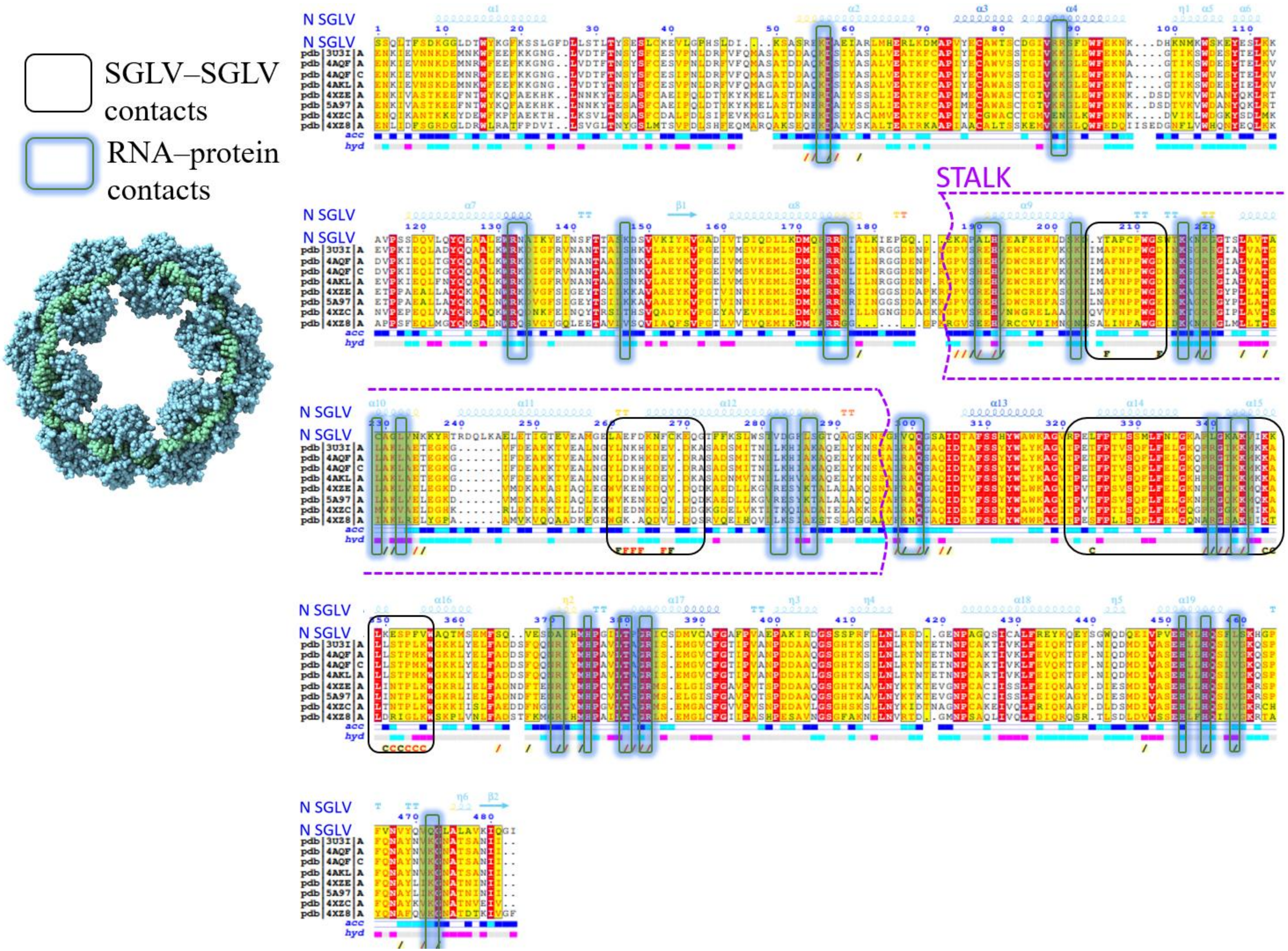
SGLV–SGLV contacts
RNA–protein contacts
STALK
N SGLV

Figure S24. AlphaFold 3 structural models of SGLV RNP hexamer complexes (top view) (**left**). In SGLV RNPs, N monomers are shown in blue, and the RNA is colored green. Contacts reported in the literature are highlighted: RNA–protein contacts are shown in blue, while SGLV–SGLV contacts are marked in black.

Sequence alignment of the SGLV N hexamer with homologous viral structures from PDB (**right**). Secondary structures are color-coded according to pLDDT, with blue indicating very high confidence and orange indicating low confidence. Below the structural annotation, MSA highlights residue conservation.

The relative accessibility (labelled 'acc') calculated by DSSP for each residue is shown with a colored bar below the sequences block: white is buried, cyan is intermediate, blue is accessible. The hydropathy (labelled 'hyd') calculated from the query sequence using the Kyte & Doolittle algorithm is shown by a second coloured bar below the accessibility: pink is hydrophobic, grey is intermediate and cyan is hydrophilic. A "/" symbol indicate that the amino acid residue in question has a contact with RNA. A red "/" symbol indicates a contact < 3.2 Å. A black "/" symbol indicates a contact between 3.2 Å and 3.7 Å.

Sequence alignment of the BJNV N hexamer with homologous viral structures from PDB (**right**). Secondary structures are color-coded according to pLDDT, with blue indicating very high confidence and orange indicating low confidence. Below the structural annotation, MSA highlights residue conservation.

The relative accessibility (labelled 'acc') calculated by DSSP for each residue is shown with a colored bar below the sequences block: white is buried, cyan is intermediate, blue is accessible. The hydropathy (labelled 'hyd') calculated from the query sequence using the Kyte & Doolittle algorithm is shown by a second coloured bar below the accessibility: pink is hydrophobic, grey is intermediate and cyan is hydrophilic. A "/" symbol indicate that the amino acid residue in question has a contact with RNA. A red "/" symbol indicates a contact < 3.2 Å. A black "/" symbol indicates a contact between 3.2 Å and 3.7 Å.

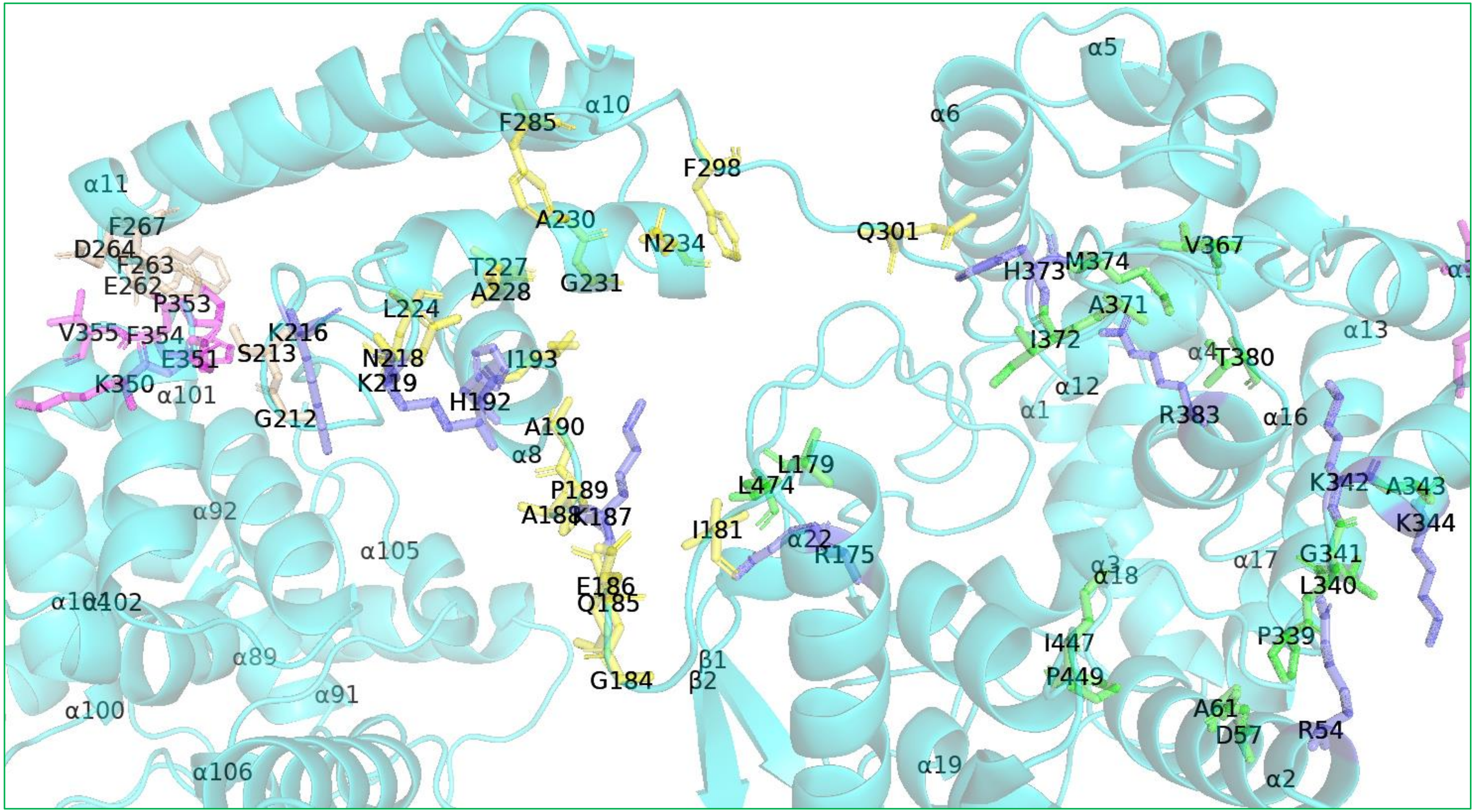

Figure S25. SGLV N from the hexameric structure. Residues from the Stalk domain in contact with ssRNA(-) (<3.5 Å) are highlighted and labeled in yellow, residues from the Head domain in contact with ssRNA(-) (<3.5 Å) are highlighted and labeled in green, and positively charged residues in both domains within the <3.5 Å contact region are additionally highlighted in blue.

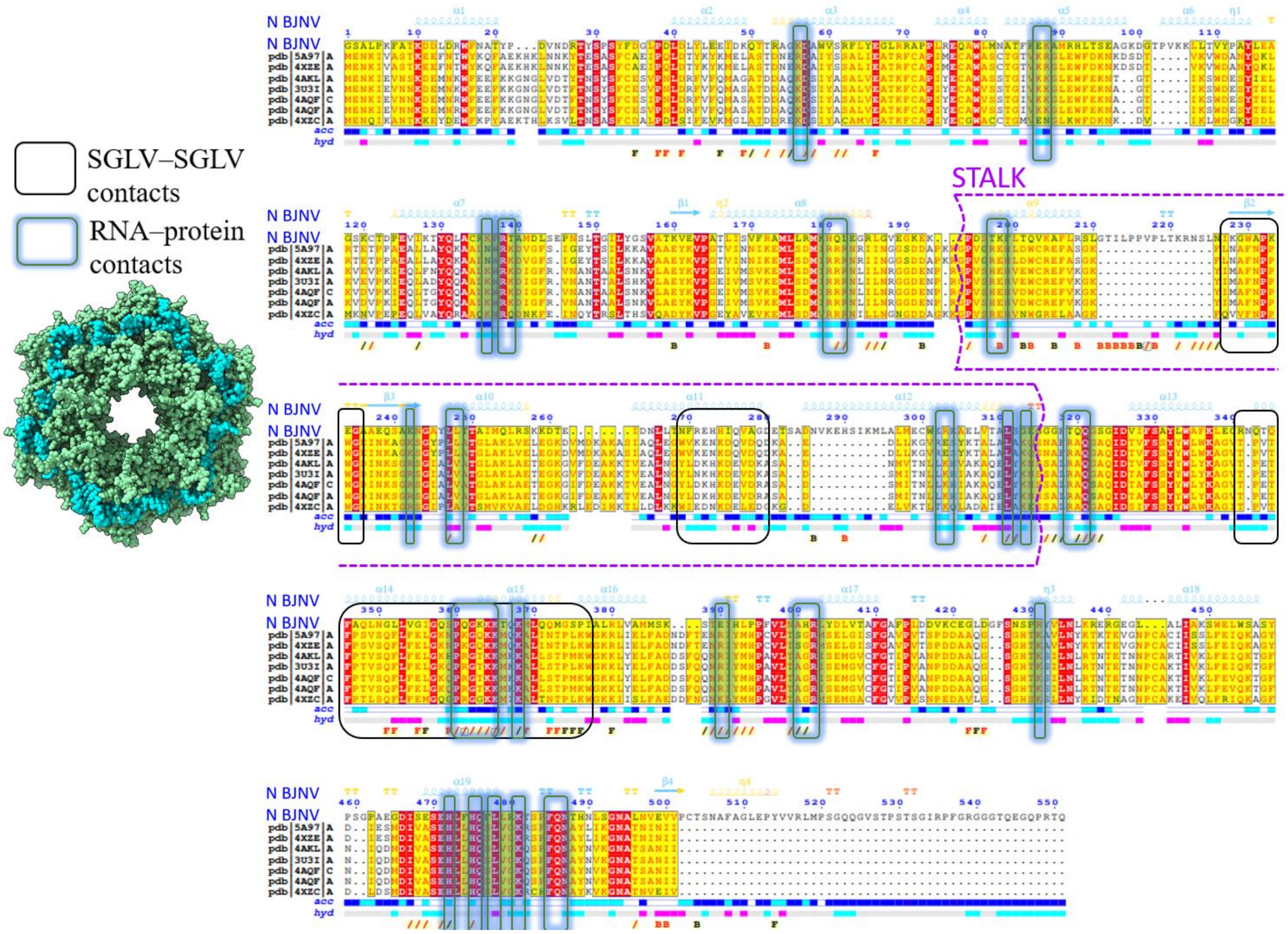
SGLV–SGLV contacts
RNA–protein contacts
STALK
N BJNV

Figure S26. AlphaFold 3 structural models of BJNV RNP hexamer complexes (top view) (**left**). In BJNV RNPs, N monomers are shown in blue, and the RNA is colored white. Contacts reported in the literature are highlighted: RNA–protein contacts are shown in blue, while BJNV – BJNV contacts are marked in black.

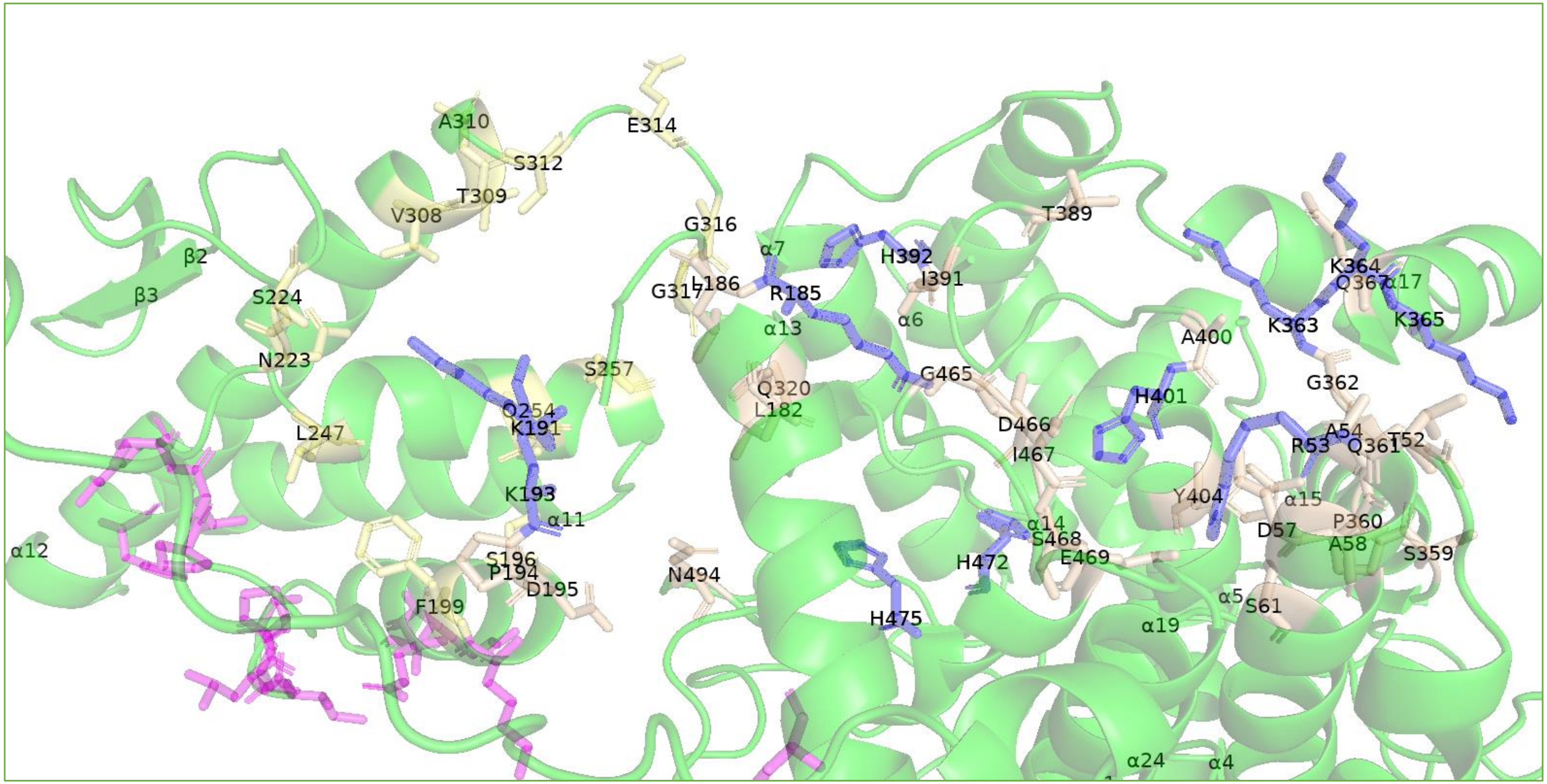

Figure S27. BJNV N from the hexameric structure. Residues from the Stalk domain in contact with ssRNA(-) (<3.5 Å) are highlighted and labeled in pale yellow, residues from the Head domain in contact with ssRNA(-) (<3.5 Å) are highlighted and labeled in pale brown, and positively charged residues in both domains within the <3.5 Å contact region are additionally highlighted in blue.

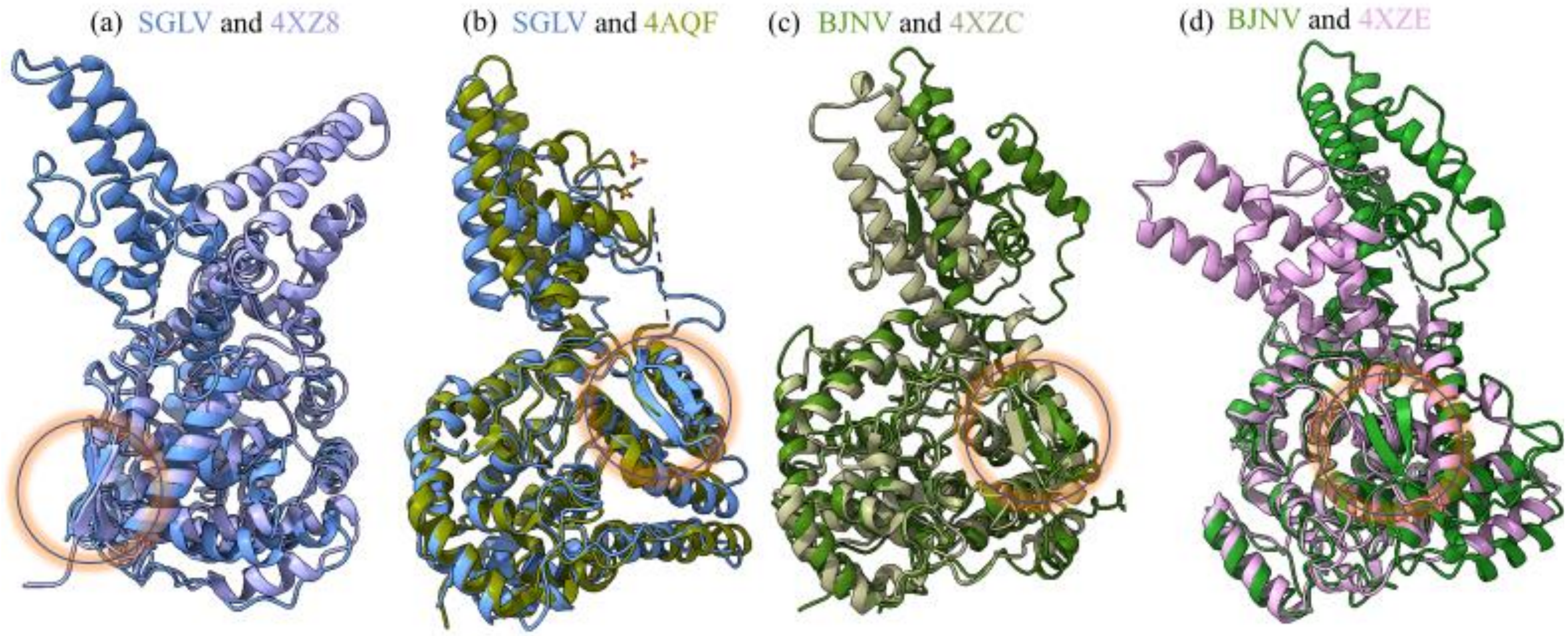

Figure S28. Imposition models of SGLV N and BJNV N tertiary structures: (a) SGLV N tertiary structure (blue) with Erve virus N (PDB ID: 4XZ8) (purple); (b) SGLV N tertiary structure (blue) with CCHFV (PDP ID: 4AQF) (green); (c) BJNV N tertiary structure (green) with Kupe virus N (PDB ID: 4XZC) tertiary structure (olive); (d) BJNV N tertiary structure (green) with Hazara virus N (PDB ID: 4XZE) tertiary structure (pink). A parallel double-stranded β-sheet found in the head domain of BJNV N and SGLV N is highlighted in the red circle.

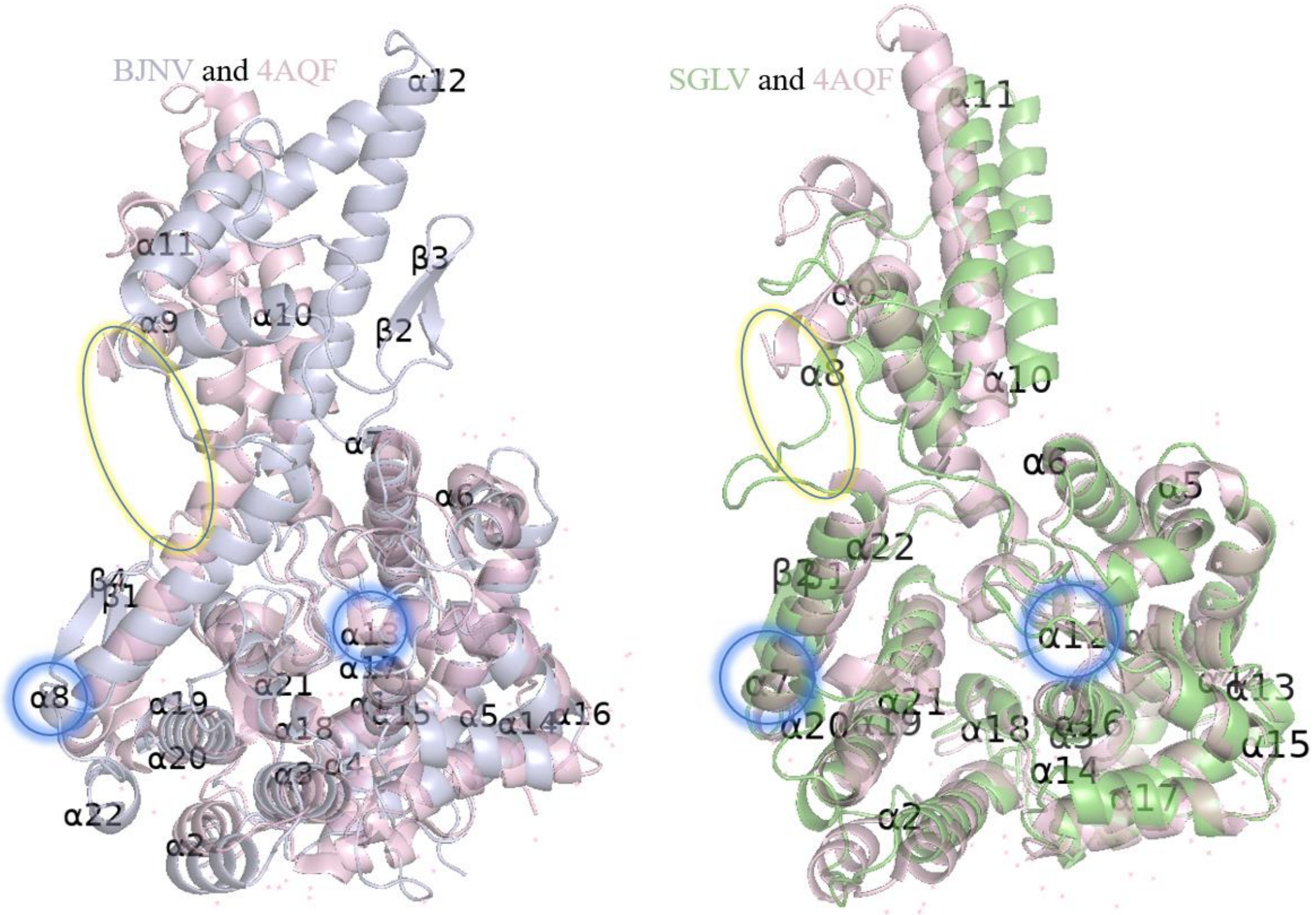

Figure S29. Imposition models of BJNV N (grey) tertiary structure with CCHFV N (pink) (PDP ID: 4AQF) (**left**). In the yellow oval the non-constructable region is highlighted, in blue - the region of connection of two domains in BJNV N.

Imposition models of SGLV N (green) tertiary structure with CCHFV N (pink) (PDP ID: 4AQF) (**right**). In the yellow oval the non-constructable region is highlighted, in blue - the region of connection of two domains in SGLV N.

Table S2. Oligonucleotides used to synthetically assemble a DNA copy of a BJNV N-coding gene

Oligonucleotide name	Oligonucleotide sequence
1BJn	CAAACCCCAACCACCCCTCCGGATCCGCACTGCCGAAA
2BJn	CCAGATCATCTTTGGTTGCAAATTTCGGCAGTGCGGATC
3BJn	TTTGCAACCAAAGATGATCTGGATCGTTGGTTTAATGCAACCTAT
4BJn	AGGTACGATCATTAACATCCGGATAGGTTGCATTAAACCAACGAT
5BJn	CCGGATGTTAATGATCGTACCTATAGTCCGAGCTATTTTGATGGT
6BJn	AGGTCCAGATCCGGCAGACCATCAAAATAGCTCGGACTAT
7BJn	CTGCCGGATCTGGACCTGTATCTGGAAGAAATTGATAAACAGAC
8BJn	CATCTTTACCTGCACGTGTGGTCTGTTTATCAATTTCTTCCAGATAC
9BJn	CACACGTGCAGGTAAAGATGCATGGGTTAGCCGTTTTCTG
10BJn	CACGACGCAGACCTTCATACAGAAAACGGCTAACCCATG
11BJn	TATGAAGGTCTGCGTCGTGCACCGCCTCTGCGTGA
12BJn	ATTCATCAGCCATGCCTGTTCACGCAGAGGCGGTG
13BJn	ACAGGCATGGCTGATGAATGCCACCTTCTTTGAAAAAGCA
14BJn	GCTGGTCAGATGACGCATTGCTTTTTCAAAGAAGGTGGC
15BJn	ATGCGTCATCTGACCAGCGAAGCAGGCAAAGATGGTACA
16BJn	GTCAGCAGCTTTTTAACCGGTGTACCATCTTTGCCTGCTTC
17BJn	CCGGTTAAAAAGCTGCTGACCGTTTATCCGGCATACCTGG
18BJn	GGTACATTTGCTACCTGCTTCCAGGTATGCCGGATAAACG
19BJn	AAGCAGGTAGCAAATGTACCGATCCGGAAGTGATTAAAACCTATC
20BJn	GAAATTTACGACATGCCAGCTGATAGGTTTTAATCACTTCCGGATC
21BJn	AGCTGGCATGTCGTAAATTTCGTACCGCAATGGATCTGAGC
22BJn	CGGTCAGGCTATTCGGTTCGCTCAGATCCATTGCGGTAC
23BJn	GAACCGAATAGCCTGACCGGTATTCTGTATGGTAGCGTTGC
24BJn	CCGGAACTTCAACTTTGGTGGCAACGCTACCATACAGAATAC
25BJn	CACCAAAGTTGAAGTTCCGGCAACACTGATTAGCGTTTTTCG
26BJn	TACGCAGCAGCATTGCACGAAAAACGCTAATCAGTGTTG
27BJn	TGCAATGCTGCTGCGTATGAAACATCAGCTGGAAGGTC
28BJn	TCTTTACCTTCAACACCCAGACGACCTTCCAGCTGATGTTTCA
29BJn	GTCTGGGTGTTGAAGGTAAAGAAAAACCGGATAGCACCAAATTT
30BJn	GAAATGCTTTAACCTGGGTCAGAAATTTGGTGCTATCCGGTTTT
31BJn	CTGACCCAGGTTAAAGCATTTCTGCGTAGCCTGGGCAC
32BJn	GGAACCGGAGGCAGAATGGTGCCCAGGCTACGCA
33BJn	CATTCTGCCTCCGGTTCCGCTGACCAAACGTAATAGTCTG
34BJn	GGTGCCCAACCTTTAATGTTCAGACTATTACGTTTGGTCAGC
35BJn	AACATTAAAGGTTGGGCACCGAAAGAAGGCGCAGCAGA
36BJn	ACCATTTTCTGCGCTCTGTTCTGCTGCGCCTTCTTTC
37BJn	ACAGAGCGCAGAAAATGGTGCATATCTGCTGACCACCG
38BJn	GCTACGCAGCTGCATAATTGCGGTGGTCAGCAGATATGC
39BJn	CAATTATGCAGCTGCGTAGCAAAAAGGATACCGAAATCGATAACC
40BJn	TCGCGAAAATTCGTCAGCAGGTTATCGATTTCGGTATCCTTTTT
41BJn	TGCTGACGAATTTTCGCGAACATCATATTCAGGTTGCCGG
42BJn	TTATCTGCGCTGGTTTCATCACCGGCAACCTGAATATGATGT

43BJn	TGATGAAACCAGCGCAGATAATGTTAAAGAACACAGCATTAAAATGC
44BJn	CAACATTCCATCAGGGCCAGCATTTTAATGCTGTGTTCTTTAACA
45BJn	TGGCCCTGATGGAATGTTGGGAACGTGAAGCAGAACTGG
46BJn	ACCGCTCAGTGCGGTAACCAGTTCTGCTTCACGTTCC
47BJn	TTACCGCACTGAGCGGTGAAGGTGGTGGTTTTACCCA
48BJn	ACATCAATACCGCTACCTTGCTGGGTAAAACCACCACCTTC
49BJn	GCAAGGTAGCGGTATTGATGTGATTTTTAGCGCATATCTGTGGG
50BJn	ATTACGGCATTCCAGTTTAAAGGCCCACAGATATGCGCTAAAAATC
51BJn	CCTTTAAACTGGAATGCCGTAATCAGACCCAGTTTGCACAGC
52BJn	CCAACCAGCAGACCATTCAGCTGTGCAAACTGGGTCTG
53BJn	TGAATGGTCTGCTGGTTGGTATTGGTCAGAGTCCGCAGG
54BJn	GTAACATTTTCTGGGTTTTCTTTTTACCCTGCGGACTCTGACCAATA
55BJn	GTAAAAAGAAAACCCAGAAAATGTTACAGCAGATGGGTAGCCCG
56BJn	AACCAGACGCAGTGCAATCGGGCTACCCATCTGCT
57BJn	ATTGCACTGCGTCTGGTTGCAATGATGAGCAAAAGCAC
58BJn	CGGTGGCAGATGAATTTCGGTGCTTTTGCTCATCATTGC
59BJn	CGAAATTCATCTGCCACCGTTTGTTCTGAATGCACATCGT
60BJn	GCGGTCACCAGATCATACAGACGATGTGCATTCAGAACAAA
61BJn	CTGTATGATCTGGTGACCGCATTTGGTGCATTTCCGCTG
62BJn	AGACCTTCGCATTTAACATCATCCAGCGGAAATGCACCAAAT
63BJn	GATGATGTTAAATGCGAAGGTCTGGATGGTTTTAGCAATAGTCCG
64BJn	CGTTTCAGATTCAGCACATAACGCGGACTATTGCTAAAACCATCC
65BJn	CGTTATGTGCTGAATCTGAAACGTGAACGTGGTGAAGGCCT
66BJn	AGCTTTTTGCAATCAGTGCCAGGCCTTCACCACGTTCA
67BJn	GGCACTGATTGCAAAAAGCTGGGAACTGTGGTCTGCAAG
68BJn	CCGGACCGCTCGGATAGCTTGCAGACCACAGTTCCC
69BJn	CTATCCGAGCGGTCCGGCAGAAGGTGATATTAGCGAAAGC
70BJn	AGAAACTGATGAAACAGGTGTTCGCTTTCGCTAATATCACCTTCTG
71BJn	GAACACCTGTTTCATCAGTTTCTGTTACGTAAAACCAGTCCGTTT
72BJn	CGCTCAGATTATGGGTATTCTGAAACGGACTGGTTTTACGTAAC
73BJn	CAGAATACCCATAATCTGAGCGGTAATGCCCTGAATGTTGAAGTT
74BJn	CATTGCTGGTACACGGAACAACTTCAACATTCAGGGCATTAC
75BJn	GTTCCGTGTACCAGCAATGCATTTGCAGGTCTGGAACC
76BJn	GGCATCAGACGAACAACATACGGTTCCAGACCTGCAAATG
77BJn	GTATGTTGTTCGTCTGATGCCGTCAGGTCAGCAGGGTGT
78BJn	GGTGCTCGGGGTGCTAACACCCTGCTGACCTGAC
79BJn	TAGCACCCCGAGCACCAGCGGTATTCGTCCGTTTG
80BJn	GTGCCACCACCACGACCAAACGGACGAATACCGCT
81BJn	GTCGTGGTGGTGGCACCCAAGAAGGTCAGCCTCG
82BJn	CTCGAGTTATTACTGTGTACGAGGCTGACCTTCTTGG
83BJn	ATACGAGCCGGAAGCATAAGATCTCGAGTTATTACTGTGTACGAGG

Table S3. Oligonucleotides used to synthetically assemble a DNA copy of a SGLV N-coding gene

Oligonucleotide name	Oligonucleotide sequence
1SLv	CAAACCCCAACCACCCCTCCGGATCCAGCCAGCTGACCTT
2SLv	GTATCCAGACCACCTTTATCACTAAAGGTCAGCTGGCTGGA
3SLv	TAGTGATAAAGGTGGTCTGGATACCTGGTACAAAGGTTTTAAAAGCAG
4SLv	TCAGCAGATCAAAACCCAGGCTGCTTTTAAAACCTTTGTACCAG
5SLv	CCTGGGTTTTGATCTGCTGAGCACCCTGACCTATAGCGA
6SLv	CAGAACTTCTTTACACAGGCTTTCGCTATAGGTCAGGGTGC
7SLv	AAGCCTGTGTAAAGAAGTTCTGGGTCCGCATAGCCTGGA
8SLv	ACGGCTTGCGCTTTTGATATCCAGGCTATGCGGACC
9SLv	TATCAAAAGCGCAAGCCGTGAAAAAGATGCAGAAATTGCACG
10SLv	TTCAGACGTTCATGCATCAGACGTGCAATTTCTGCATCTTTTTC
11SLv	TCTGATGCATGAACGTCTGAAAGATATGGCACCGGTTTATGA
12SLv	CAGCTGGTCCATGCACATTCATAAACCGGTGCCATATCT
13SLv	ATGTGCATGGACCAGCTGTGATGGTATTGTTCGTCGTAGC
14SLv	CTTTGTTTTTCTCGAACCAATCAAAGCTACGACGAACAATACCATCA
15SLv	TTTGATTGGTTCGAGAAAAACAAAGACCACAAAAACATGAAATGGTCC
16SLv	CTTTTTCAGGCTCTCATACTCTTTGGACCATTTCATGTTTTTGTGGT
17SLv	AAAGAGTATGAGAGCCTGAAAAAGGCAGTTCCGAGCAGCG
18SLv	TCTTGATACTGCAGAACCTGATCGCTGCTCGGAACTGC
19SLv	ATCAGGTTCTGCAGTATCAAGAGGCAGCACTGGAATGGC
20SLv	CTGTTGATTTCATATTTGATTGCATTACGCCATTCCAGTGCTGCC
21SLv	GTAATGCAATCAAATATGAAATCAACAGCTTTACCACCGCCAGCAA
22SLv	ACGATAGATTTTAACAACGCTATCTTTGCTGGCGGTGGTAAAG
23SLv	AGATAGCGTTGTTAAAATCTATCGTGTGGGTGCAGATATTGTGAC
24SLv	TTCAGCAGGTCCTGAATATCGGTCACAATATCTGCACCCAC
25SLv	CGATATTCAGGACCTGCTGAAAGACATGCAGCGTCGTCG
26SLv	GGTTCAATTTTCAGTGCGGTATTACGACGACGCTGCATGTCT
27SLv	TAATACCGCACTGAAAATTGAACCGGGTCAAGAAAAAGCACCG
28SLv	CTTTAAATGCTTCAATATGCAGTGCCGGTGCTTTTTCTTGACCC
29SLv	GCACTGCATATTGAAGCATTTAAAGAATGGCTGGACAGCAAAGA
30SLv	GGACACGGTGCGGTATAGTCTTTGCTGTCCAGCCATT
31SLv	CTATACCGCACCGTGTCCGTGGGGTAGCTGGACCA
32SLv	GCTTGTACCGCCTTTATTCTTTTTGGTCCAGCTACCCCAC
33SLv	AAAAGAATAAAGGCGGTACAAGCCTGGCAGTTACCGCATGT
34SLv	CGATATTTCTTATTAACCAGACCTGCACATGCGGTAACTGCCAG
35SLv	GCAGGTCTGGTTAATAAGAAATATCGTACCCGTGATCAGCTGAAAG
36SLv	GCCAATGGTTTCCAGTTCTGCTTTCAGCTGATCACGGGTA
37SLv	CAGAACTGGAAACCATTGGCACCGAAGTTGAAGCAATGGG
38SLv	TTTTTATCGAATTCTGCCAGTTCACCCATTGCTTCAACTTCGGT

39SLv	TGAACTGGCAGAATTCGATAAAAACTTTTGTAAAGAACAGGGCAC C
40SLv	GTTGACCACAGACTTTTGAAGAAGGTGCCCTGTTCTTTACAAAAG
41SLv	TTCTTCAAAAGTCTGTGGTCAACCGTTGATGGTTTTCTGAGCG
42SLv	CTACCTGCCTGGGTGCCGCTCAGAAAACCATCAACG
43SLv	GCACCCAGGCAGGTAGCAAAAATAGCGGTTTTGTTCAGC
44SLv	GGTATCAATTGCGCTACCCTGCTGAACAAAACCGCTATTTTTG
45SLv	AGGGTAGCGCAATTGATACCGCATTTAGCAGCCATTATTGGG
46SLv	CGAACACCGGCTTTCCATGCCCAATAATGGCTGCTAAATGC
47SLv	CATGGAAAGCCGGTGTTCGTCCGGAACTGTTTCCGACA
48SLv	AGATTAAACAGCATACTGCTCAGTGTCGGAAACAGTTCCGGA
49SLv	CTGAGCAGTATGCTGTTTAATCTGGGTAAAGCACCGCTGG
50SLv	CAGTTTCTTAATAACTTTTGCTTTGCCCAGCGGTGCTTTACCC
51SLv	GCAAAGCAAAAGTTATTAAGAAACTGAAAGAAAGCCCGTTTGTTT GG
52SLv	TTTCGCTCATGGTCTGTGCCCAAACAAACGGGCTTTCTTT
53SLv	GCACAGACCATGAGCGAAATGTTTAGCCAGGTTGAAAGTGA
54SLv	CCGGATGCATATGAATGGCATCACTTTCAACCTGGCTAAACA
55SLv	TGCCATTCATATGCATCCGGGTATTCTGACACCGGGTCG
56SLv	GCACAAACCATATCGCTACAAATACGACCCGGTGTCAGAATAC
57SLv	TATTTGTAGCGATATGGTTTGTGCATTTGGTGCATTTCCGGTTG
58SLv	ACGAATTTTTGCCGGTTCTGCAACCGGAAATGCACCAAAT
59SLv	CAGAACCGGCAAAAATTCGTGATGGTAGCAGCAGTCCG
60SLv	CGCAGATTCAGCAGAAAACGCGGACTGCTGCTACCATC
61SLv	CGTTTTCTGCTGAATCTGCGTAGTGATGGTGAAAATCCGGC
62SLv	TGCACAAATGCTCTGACCTGCCGGATTTTCACCATCACTA
63SLv	AGGTCAGAGCATTTGTGCACTGTTTCGTGAATATAAACAAGAGTAT AG
64SLv	TTCTTGATCCTGCCAACCGCTATACTCTTGTTTATATTCACGAAACA G
65SLv	CGGTTGGCAGGATCAAGAAATTGTTCCGGTTGAACATATGC
66SLv	GCTCAGAAAGCTCTGATGCAGCATATGTTCAACCGGAACAAT
67SLv	TGCATCAGAGCTTTCTGAGCAAACATGGTCCGTTTGTTAATGT
68SLv	GCCAGACCTTGAACCTGATACACATTAACAAACGGACCATGTTT
69SLv	GTATCAGGTTCAAGGTCTGGCACTGGCCGTTAAAATTCAGG
70SLv	ATACGAGCCGGAAGCATAAGATCTCGAGTCATTAAATACCCTGAA TTTTAACGGCCAG

Table S4. The pET28b-14xHis cloning primers

Oligonucleotide name	Oligonucleotide sequence
1BJn	CAAACCCCAACCACCCCTCCGGATCCGCACTGCCGAAA
83BJn	ATACGAGCCGGAAGCATAAGATCTCGAGTTATTACTGTGTACGA GG
1SLv	CAAACCCCAACCACCCCTCCGGATCCAGCCAGCTGACCTT
70SLv	ATACGAGCCGGAAGCATAAGATCTCGAGTCATTAAATACCCTGA ATTTTAACGGCCAG

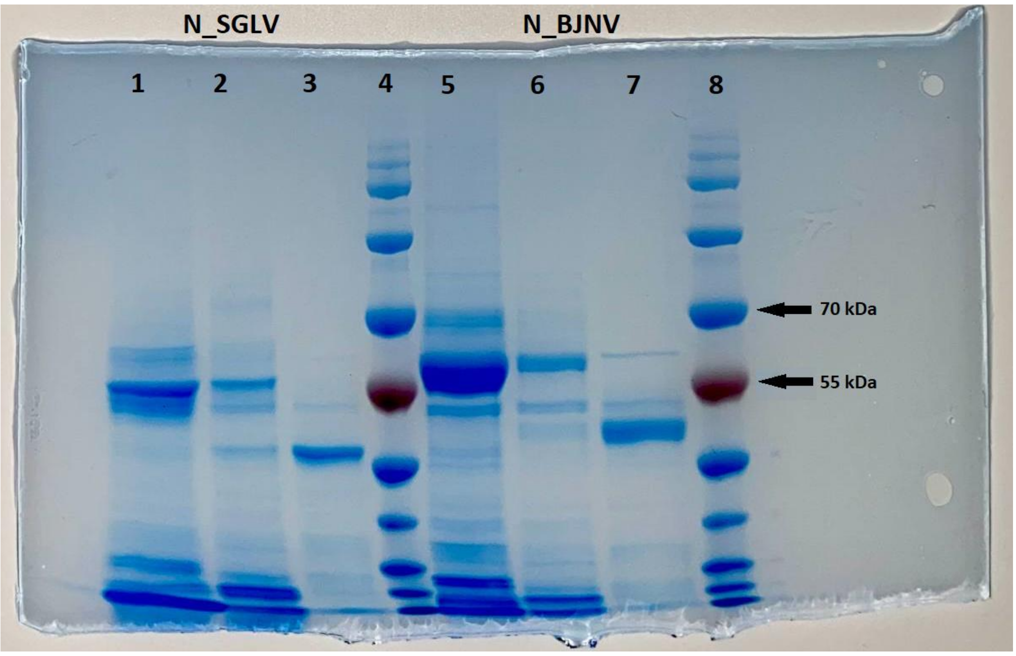

Figure S30. SDS-PAGE electropherogram of BJNV N and SGLV N preparations prior to exclusion chromatography:

1. SGLV N fraction after IMAC I, before affinity tag cleavage;
2. SGLV N fraction after hydrolysis;
3. SGLV N fraction after IMAC II;
4. Molecular weight marker;
5. BJNV N fraction after IMAC I, before affinity tag cleavage;
6. BJNV N fraction after hydrolysis;
7. BJNV N fraction after IMAC II;
8. Molecular weight marker.

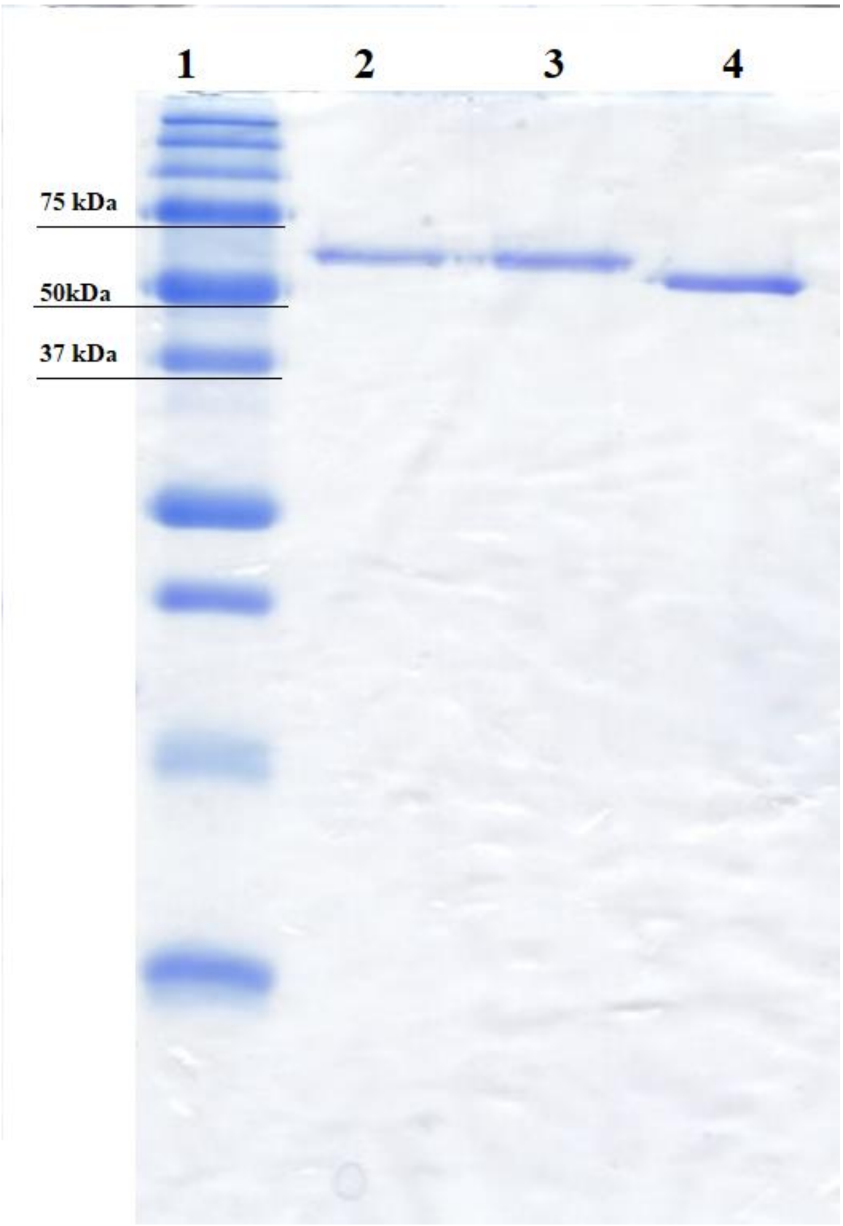

Figure S31. SDS-PAGE electropherogram of BJNV N and SGLV N preparations after SEC:
1. Molecular weight marker;
2. BJNV N fraction 1 after SEC;
3. BJNV N fraction 2 after SEC;
4. SGLV N fraction after SEC.
